# *N-*acetylmuramic acid recognition by MurK kinase from the MurNAc auxotrophic oral pathogen *Tannerella forsythia*

**DOI:** 10.1016/j.jbc.2023.105076

**Published:** 2023-07-20

**Authors:** Aleksandra Cecylia Stasiak, Karolin Gogler, Marina Borisova, Phillipp Fink, Christoph Mayer, Thilo Stehle, Georg Zocher

**Affiliations:** 1 Interfaculty Institute of Biochemistry, University of Tuebingen, Tuebingen, Germany; 2 Interfaculty Institute of Microbiology and Infection Medicine, Organismic Interactions/Glycobiology, University of Tuebingen, Tuebingen, Germany

**Keywords:** ATPase, bacterial metabolism, cell wall, cell wall recycling, MurNAc kinase, periodontal disease, protein structure

## Abstract

The bacterial cell wall consists of a three-dimensional peptidoglycan layer, composed of peptides linked to the sugars *N*-acetylmuramic acid (MurNAc) and GlcNAc. Unlike other bacteria, the pathogenic *Tannerella forsythia*, a member of the red complex group of bacteria associated with the late stages of periodontitis, lacks biosynthetic pathways for MurNAc production and therefore obtains MurNAc from the environment. Sugar kinases play a crucial role in the MurNAc recycling process, activating the sugar molecules by phosphorylation. In this study, we present the first crystal structures of a MurNAc kinase, called murein sugar kinase (MurK), in its unbound state as well as in complexes with the ATP analog β-γ-methylene adenosine triphosphate (AMP-PCP) and with MurNAc. We also determined the crystal structures of K1058, a paralogous MurNAc kinase of *T. forsythia*, in its unbound state and in complex with MurNAc. We identified the active site and residues crucial for MurNAc specificity as the less bulky side chains of S133, P134, and L135, which enlarge the binding cavity for the lactyl ether group, unlike the glutamate or histidine residues present in structural homologs. In establishing the apparent kinetic parameters for both enzymes, we showed a comparable affinity for MurNAc (K_m_ 180 μM and 30 μM for MurK and K1058, respectively), with MurK being over two hundred times faster than K1058 (V_max_ 80 and 0.34 μmol min^−1^ mg^−1^, respectively). These data might support a structure-guided approach to development of inhibitory MurNAc analogs for pathogen MurK enzymes.

*Tannerellaforsythia* is a Gram-negative oral pathogen that is part of the red complex of bacterial species associated with late stages of periodontitis, an inflammatory disease of gums and bones supporting teeth (1). Like other bacteria, *T. forsythia* possesses a three-dimensional peptidoglycan (PGN) network in the cell wall, consisting of N-acetylmuramic acid (MurNAc) and GlcNAc sugars and peptides that are connected by glycosidic, peptide, and amide bonds (2). Unlike other bacteria, however, the organism depends on MurNAc as a growth factor (3) (except for some canine and feline isolates (4)), since it is unable to synthesize MurNAc due to the absence of biosynthetic genes (5). To maintain its PGN layer, *T. forsythia* relies on scavenging MurNAc from the environment (6, 7), which involves the action of periplasmic *N-*acetylmuramidases (8), the sugar transport across the cell membrane (7, 9), and the synthesis of UDP-MurNAc *via* an uridylyltransferase (10). The *T. forsythia* ATP-dependent murein sugar kinase (MurK, NCBI Reference Sequence: WP_046825531.1) has previously been identified as a part of the Tf_*murTKQ* operon (Fig. 1) coding for enzymes responsible for import and cytoplasmic processing of MurNAc (11). MurK was shown to have a strong kinetic preference for MurNAc, yielding MurNAc 6-phosphate (MurNAc-6P; see Fig. 1), though with the ability to phosphorylate GlcNAc, albeit with very low affinity and reaction rate (11). As it is genetically linked to the MurNAc 6-phosphate etherase (MurQ), which converts MurNAc-6P to GlcNAc 6-phosphate, its apparent role is mainly to utilize MurNAc as a GlcNAc source rather than to yield UDP-MurNAc to sustain cell wall synthesis. Consistent with this view, a *T. forsythia murK* deletion mutant was shown to block MurNAc catabolism and allow the direction of MurNAc solely to PGN biosynthesis, resulting in a growth advantage in MurNAc-depleted medium (11). In addition to MurK, a second putative MurNAc kinase–encoding gene (NCBI Reference Sequence: WP_041590480.1) was identified in the genome of *T. forsythia*. This kinase, provisionally referred to as K1058 in this work, is encoded by a gene (*Tanf_RS08300*) located directly upstream of the PGN-recycling gene cluster located directly upstream of the Tf_*murTKQ* operon (12) and shares 44% sequence identity with MurK (Fig. S1, Table S1).

**Figure 1:**
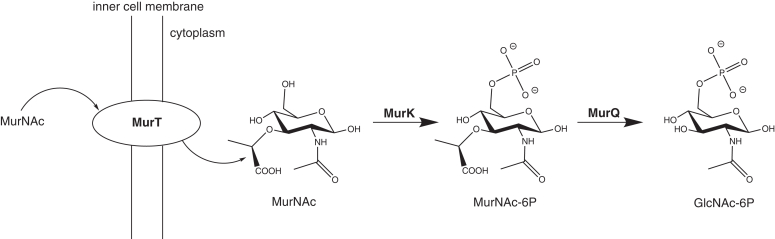
**The MurTQK pathway in *Tannerella forsythia*: extracellular MurNAc is transported into the cytoplasm by MurT.** MurNAc phosphorylation to MurNAc-6P is catalyzed by kinases MurK and K1058 (the relation of the latter to the MurTQK pathway is unclear), using ATP as a phosphate group donor. MurNAc-6P is converted to GlcNAc-6-P by MurQ. MurK, murein sugar kinase; MurNAc, N-acetylmuramic acid; MurNAc-6P, N-acetylmuramic acid-6 phosphate.

Sugar kinases catalyze the activation of sugar molecules by phosphorylation, commonly though not exclusively using ATP as the terminal phosphate group donor (13), and have been divided into five structural classes: hexokinases, repressor-ORF kinases (ROKs), ribokinases, GHMP (galactokinase, homoserine kinase, mevalonate kinase, and phosphomevalonate kinase) kinases, and phosphatidylinositol kinases (14). All these families employ the same reaction mechanism, where a conserved aspartic acid residue deprotonates the hydroxyl group, which then forms a transient pentavalent state with the ATP molecule, before the ADP moiety leaves. The presence of divalent metal ions such as magnesium or zinc is also a common requirement (14). In addition to phosphorylating glucose, glycokinases have many other sugar substrates, such as fructose, *N-*acetylmannosamine, GlcNAc, allose (13, 15), or even more complex glycan molecules such as muramyl dipeptides. Recently, the human GlcNAc kinase *N-*acetylglucosamine kinase (NagK) was shown to act as a MurNAc kinase that specifically phosphorylates muramyl dipeptide and thereby activates this immunogenic ligand for bacterial recognition *via* the nucleotide-binding oligomerization domain-containing protein 2 (NOD2) receptor (16). Besides their role in primary metabolism, sugar kinases also catalyze secondary metabolism reactions, generating products necessary for other pathways (15). In bacteria, such reactions enable the organism to produce molecules for cell wall synthesis or reshaping, crucial in the formation of the barrier between the environment and the organism (5, 17, 18, 19).

MurNAc-specific kinases have not yet been structurally characterized, although previous work has functionally characterized the MurNAc- and GlcNAc-specific MurK from *Clostridium acetobutylicum* (20). Here, we present high-resolution crystal structures of MurK as an apo enzyme, in complex with an ATP analog, and in complex with its MurNAc substrate, based on which we model the ternary complex to describe the mode of enzymatic catalysis. To compare MurK and K1058, we expanded our investigation to structures of K1058 in its apo and MurNAc-bound forms. Structural and sequence data showed that MurK and K1058 both belong to the group of ribonuclease H-like kinases and are members of the ASKHA (acetate and sugar kinase/hsc70/actin) superfamily (21). The conserved catalytic aspartate residue is present in both enzymes. Based on the data, we identified a nonconserved patch potentially important for MurNAc recognition in MurK. To gain insight into their roles in the cell wall metabolism in *T. forsythia*, we established the apparent kinetics of both enzymes for their ability to turn over MurNAc.

## Results

### Structure determination of MurK and K1058

MurK and K1058 are two sugar kinases of the cell wall recycling system of *T. forsythia*, sharing a sequence identity of 44% (Fig. S1, Table S1) and separated by a single PGN-scavenging operon within the genome. To investigate the relationship between this sequence similarity, we determined the structure of both enzymes. We produced and purified MurK and K1058 by recombinant expression as His_6_-tagged proteins in *Escherichia coli*. Both kinases eluted as highly pure, dimeric enzymes from a size-exclusion chromatography (SEC) column with an apparent molecular mass of approximately 60 kDa (molecular mass for MurK and K1058 is 32.4 and 32.3 kDa, respectively). Both enzymes were successfully crystallized. We obtained triclinic crystals for MurK (termed MurKapo), which contain four protomers in the asymmetric unit (ASU) that form two biological dimers. K1058 crystallized in a tetragonal space group (termed K1058apo) with a single protomer in the ASU. The biological corresponding dimer interface as is observed for MurKapo results from the application of the crystallographic two-fold axis. To obtain insight into substrate binding and catalysis of the kinases, we determined substrate-bound structures of MurK and K1058. Initial attempts of soaking MurNAc into MurKapo or K1058apo crystals with or without an ATP analog failed, probably due to an induced fit accompanying MurNAc binding. Instead, we pursued cocrystallization with MurNAc and/or β-γ-methylene adenosine triphosphate (AMP-PCP), a less hydrolyzable analog of the phosphate donor ATP. We successfully cocrystallised MurK with AMP-PCP (termed MurK/AMP-PCP) and established its structure at 2.12 Å resolution. Crystals of MurK/AMP-PCP were monoclinic and contain a biological dimer in the ASU. The production of MurNAc-bound crystals of MurK and K1058 was very challenging but eventually yielded crystals of MurK and K1058 in complex MurNAc (termed MurK/MurNAc and K1058/MurNAc, respectively). Both crystal forms diffract to approximately 3 Å resolution and contain six protomers in the ASU corresponding to three biological dimers. Crystals of MurK/MurNAc suffered from perfect merohedral twinning.

**Figure 2:**
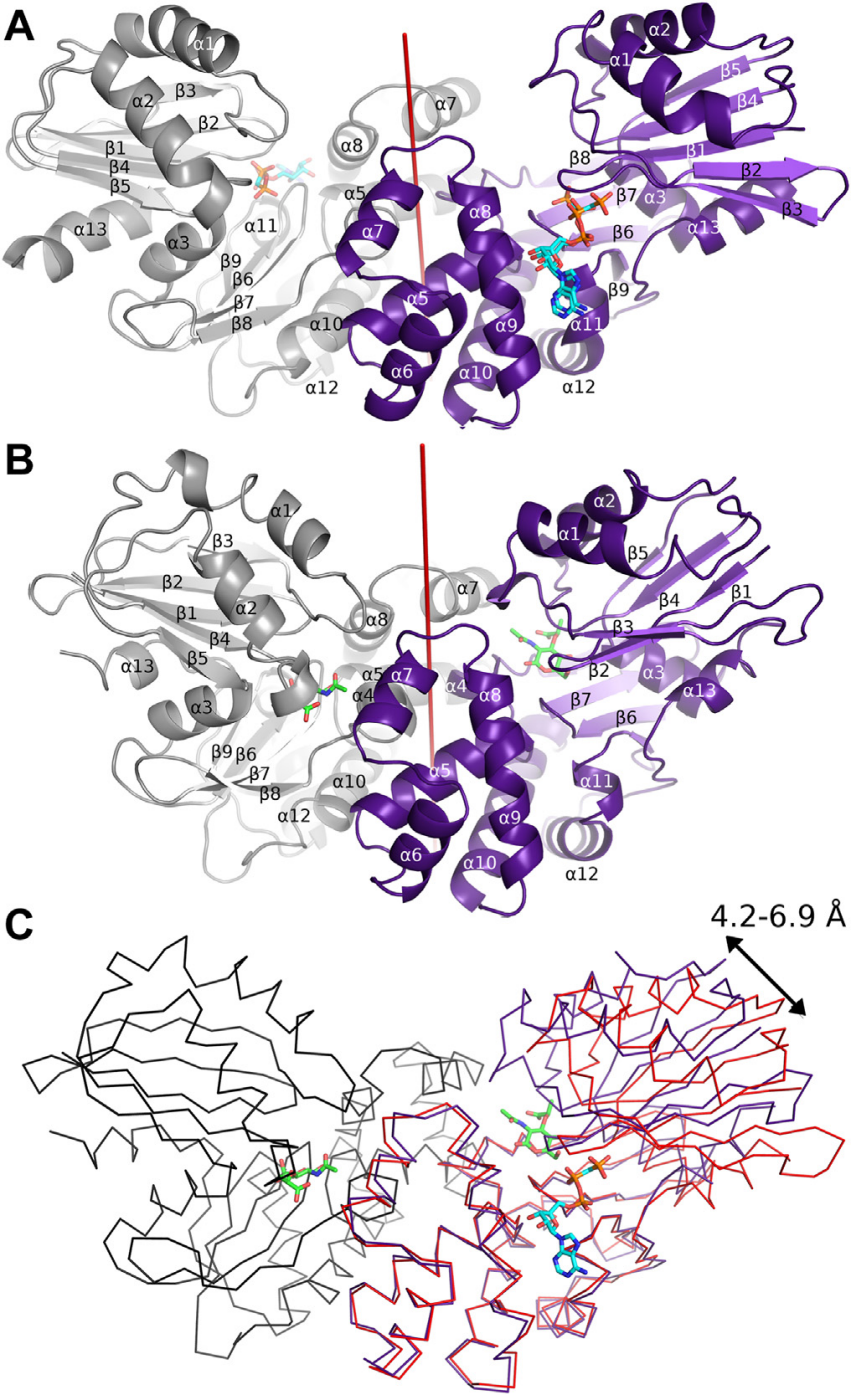
**The structures of *Tannerella forsythia* MurK in complex with its substrates.***A*, cartoon representation of the structure of MurK as a dimer in complex with the ATP analog AMP-PCP (*blue* and *orange*). Two conformations are shown for AMP-PCP in one of the chains, differing mainly in the position of the terminal phosphoryl group. One of the chains is shown in *gray*, the other in *purple* (chain B). *B*, cartoon representation of the structure of MurK as a dimer in complex with the MurNAc substrate (*green*). Color scheme as in (*A*). *C*, movement of MurK on binding the sugar substrate: ribbon representation of the ATP analog-bound MurK (*red*) and MurNAc-bound MurK (*purple*). The smaller N-terminal domain shifts into a closed conformation by 4.5 to 6.9 Å. The other chain of the MurK/MurNAc dimer is showed as *black ribbon* for reference. MurK, murein sugar kinase; MurNAc, N-acetylmuramic acid.

**Figure 3:**
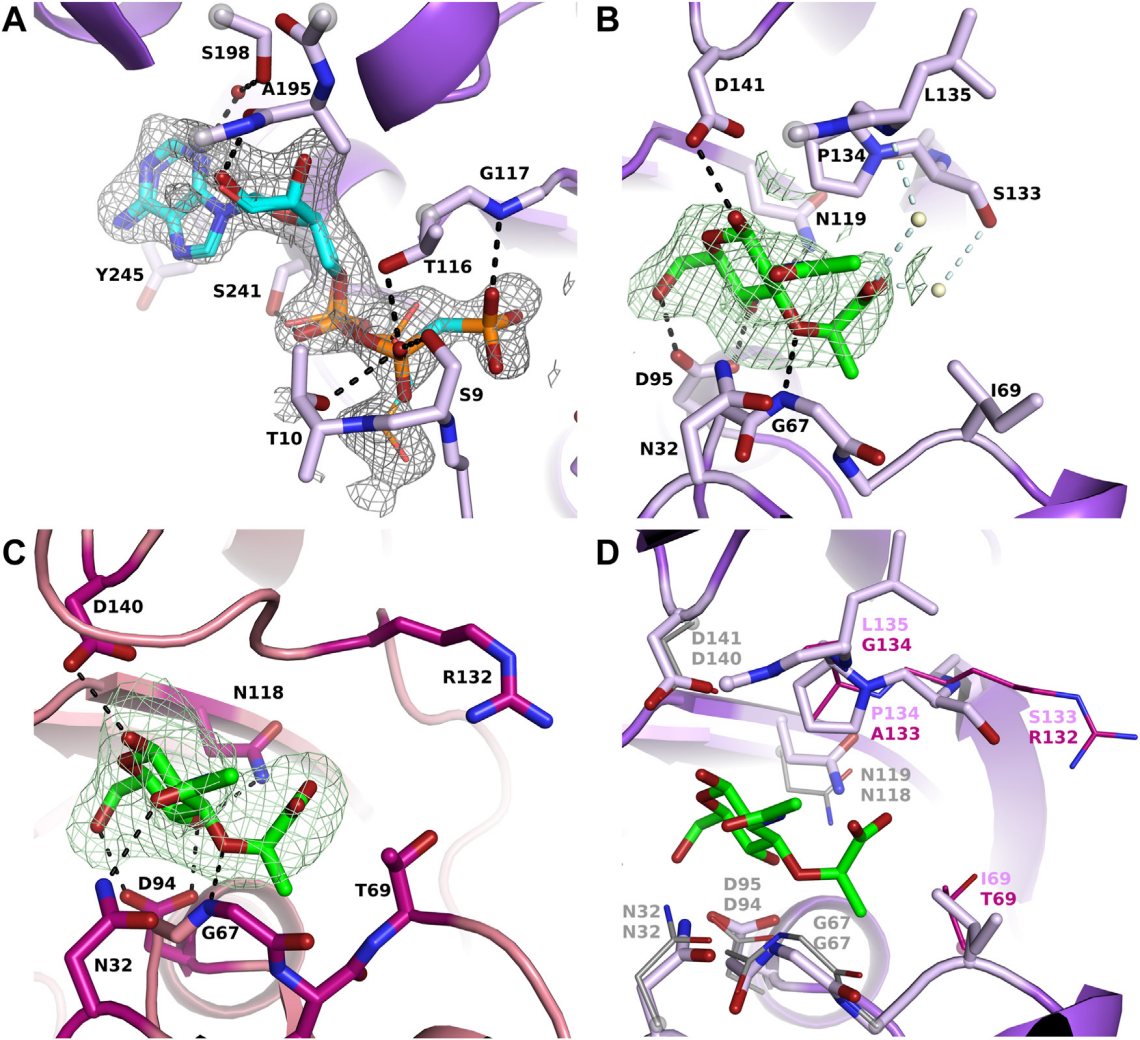
**Substrate-binding sites of MurK and K1058.** Binding sites of MurK with AMP-PCP bound (*A*), MurK with MurNAc bound (*B*), and K1058 with MurNAc bound (*C*). Residues presumed to be involved in binding and substrates (MurNAc, *green*; AMP-PCP, *blue* and *orange*) are shown as *sticks*. Alternative conformation of AMP-PCP, pointing away from the MurNAc-binding site, is shown as lines. *Black dashes* represent proposed hydrogen bonds. *Red spheres* represent water molecules. In (*B*), *pale blue dashes* and *ivory spheres* represent water molecules and water-mediated hydrogen bonds postulated only based on their presence in MurNAc/AMP-PCP structure. Density from the simulated annealing Fo-Fc omit map shown as mesh at 3σ (both MurNAc structures) or 2.5σ (MurK/AMP-PCP structure). Best-quality density is chosen from multiple chains, and density in other chains is shown in Fig. S6. *D*, superposition of the MurK and K1058 MurNAc-binding sites. MurK residues are shown as *purple sticks*, and K1058 residues are shown as *lines*. Only amino acids forming hydrogen bonds or differing between the binding sites of the two proteins are shown. Residues which are conserved between the two sites are shown in *gray* for K1058, and different residues are shown in *magenta*. Helices α3, α8, α9, α12, α13, beta sheet β9 and β10, loops β1β2, α7α8 (*A*), helices α3, α4, α10, sheets β6, β7, β8, and loops β3α1, α10β9 (*B*), helices α1, α4, sheets β6, β7, β8, and loops β7β8, α10β9, α12α13 (*C*), helices α3, α4, α13, sheets β6, β7, β9, loop β3α1, α3b6, β7β8, α10β9, α12α13 (*D*) have been partly or completely removed for clarity. MurK, murein sugar kinase; MurNAc, N-acetylmuramic acid.

Although, both substrates (AMP-PCP and MurNAc) were used to produce MurK/MurNAc and K1058/MurNAc crystals, a ternary complex could not be established, probably due to a very long crystallization time (6–9 months, in contrast to 3 days for the MurK/AMP-PCP crystals), during which the ATP analog seems not to have remained stable in the crystallization conditions. X-ray data showed continuous electron density for both kinases and allowed tracing of the complete protein chains in all structures (Figs. 2 and S2) except for the final C-terminal residues (for K1058 and MurK at least residues 1–277). The electron density for AMP-PCP and MurNAc in the corresponding structures unequivocally identified both ligands and allowed for placing of the compounds with full occupancy into the active sites of all chains of the enzymes (Fig. 3), beside MurK/MurNAc. Here, two chains showed weak electron density probably due to merohedral twinning, which allowed the sugar to be placed only in four out of six chains of the ASU.

In summary, we established the unliganded structures of MurK (MurKapo) and K1058 (K1058apo), a MurK structure with the phosphate analog AMP-PCP (MurK/AMP-PCP), and structures of both MurK and K1058 with MurNAc (MurK/MurNAc and K1058/MurNAc, respectively). Data collection and model quality criteria of all structures are summarized in Table 1.

**Table 1:** Data collection and refinement statistics for MurK and K1058 structures

Data collection	MurKapo	MurK/AMP-PCP	MurK/MurNAc	K1058apo	K1058/MurNAc
Space group	P1	P 2_1_	I4∗	I 4_1_ 2 2	P 3_1_ 2 1
Cell dimensions					
*a*, *b*, *c* (Å)	58.76, 59.51, 96.71	61.70, 55.65, 86.00	113.43, 113.43, 294.76	131.94, 131.94, 89.22	143.75, 143.75, 210.95
*α,β*,γ (°)	74.30, 75.27, 65.95	90, 93.60, 90	90, 90, 90	90, 90, 90	90, 90, 120
Resolution (Å)	49.50-2.05 (2.05-2.17)	48.6-2.12 (2.12-2.18)	49.13-2.7 (2.87-2.7)	49.22-2.00 (2.00-2.05)	29.85-3.06 (3.14-3.06)
No. of unique reflections	68,635 (11,045)	33,324 (2435)	49,814 (8018)	26,844 (1946)	48,103 (3521)
Redundancy	2.4 (2.4)	6.8 (7.0)	14.1 (14.5)	26.6 (26.2)	23.6/20.5
*R*_*meas*_*(%)*	9.7 (65.3)	17.9 (141.6)	40.8 (338)	18.8 (267.2)	45.6/(331.3)
*I*/σ(*I)*	9.1 (1.9)	10.4 (1.5)	8.1 (1.2)	19.6 (1.5)	8.1 (1.0)
Completeness (%)	96.1 (95.7)	100 (100)	98.5 (98.6)	100 (100)	99.9/100
*CC*_*1/2*_	99.7 (75.1)	99.6 (57.4)	99.4 (46.6)	99.9 (53.4)	99.3 (34.8)
*Wilson B*-factor (Å^2^)	38	36	65	40	62
Refinement					
*R*_work_/*R*_free_	0.207/0.254	0.185/0.233	0.248/0.277	0.177/0.206	0.235/0.259
*B*-factors (Å^2^)					
Chain A/B/C/...	34/35/39/42	36/33	72/48/72/51/71/99	42	74/68/73/113/103/92
Ligand	-	58/34	66/40/-/48/61/-	-	62/57/58/105/78/76
RMS deviations					
Bond lengths (Å)	0.003	0.011	0.009	0.007	0.001
Bond angles (°)	1.06	1.79	1.85	1.46	0.366
Ramachandran					
Favored [%]	94.6	94.2	90.8	96.4	94.1
Outliers [%]	0.6	1.1	1.9	0.7	0.4

Asterisk (∗) denotes a twinned structure.

### Overall architecture of MurK kinases

According to the classification by Cheek *et al.* (21), the kinase MurK belongs to the group of ribonuclease H-like kinases and is a member of the ASKHA (acetate and sugar kinase/hsc70/actin) superfamily. MurK is a member of the glucokinase family of hexokinases that phosphorylate their conjugate sugar substrate at the opposite site of the anomeric C-atom (14), which is the hydroxyl group at atom C6 of MurNAc (Fig. 1). Structurally, MurK possesses the glucokinase fold (Figs. 2, *A* and *B* and S3), sharing the same topology characterized by a small N-terminal α/β domain (residues 1–104) and a large C-terminal α/β domain (residues 104–284). The core motif of both domains resembles the core motif of the ribonuclease H fold, a three-layered α/β/α-motif with βββαβαβα topology, where the second β-strand runs antiparallel to the central β-sheet. In contrast to the N-terminal core motif, seven α-helices (α4 to α10) are inserted into the larger C-terminal domain of the enzyme after the third β-strand β8. These insertions contain the majority of amino acids that constitute the two-fold symmetrical dimeric interface of the MurK.

In agreement with other glucokinases (22), a detailed analysis of all three crystal lattice systems observed for MurK using Evolutionary Protein Protein Interface Classifier (EPPIC) and Protein Data Bank Proteins, Interfaces, Structures and Assemblies (PDB PISA; (23, 24)) revealed a dimeric assembly of the enzyme (Fig. 2, *A* and *B*), which was confirmed by SEC experiments. A detailed C_α_-RMSD analysis of all four chains in MurKapo showed that both protomers of a dimer possess a virtually identical conformation that is slightly different to the second dimer of the ASU (Fig. S4). The dimerization interface of the apo enzyme buries a surface of around 1160 Å^2^ and involves just two residues (F34 and F35) of the N-terminal domain, whereas all other contacts to the adjacent protomer were made by several locations on the α-helical insertion of the C-terminal domain. These include residues in the helices α4-α9 and the loop region in between β8 and α4, as well as residues in the loop region α10 to β9. The interactions of the dimeric assembly are detailed in Tables S2–S4. The dimer buried surface area increased to 1600 to 1700 Å^2^ for MurNAc-bound structures, an effect that is also reported for the human *N-*acetylmannosamine kinase by Martinez *et al.* (25).

### Comparison to other sugar kinases

As expected, a search for structurally related proteins (26) revealed similarities to glucokinases (some not biochemically characterized), ROK hexokinases, but also to actin-related proteins with C_α_-RMSDs ranging from 1.2 Å to around 4.2 Å. We focused our comparison on sugar kinases. Here, the closest structural homologs to MurK are two proteins, both targets of the Northeast Structural Genomics project (PDB ID 1ZXO and 1ZBS), both possessing a very low C_α_-RMSD of 1.1 Å in relation to MurKapo (Table S5). These proteins are not biochemically characterized, and their natural substrates are therefore unknown; however, 1ZBS has been described as a putative GlcNAc kinase. Based on structural similarity, it is possible that MurNAc would be a substrate candidate. All other related sugar kinases are structurally less well conserved, with C_α_-RMSD values above 2.8 Å, which is the C_α_-RMSD for human GlcNAc kinase (22), the closest hit for which the substrate specificity is known and for which complex structures with ADP and GlcNAc were also established. In comparison to the human GlcNAc kinase, a member of the glucokinase family, the C terminus of MurK is substantially shortened. For detailed comparison of the active site, we focused on hits with established substrate specificities and structurally characterized sugar binding.

### Induced fit of MurK kinase upon substrate binding

Soaking experiments with MurKapo crystals using MurNAc failed, probably due to a rearrangement of the N-terminal domain upon substrate uptake ultimately leading to severe crystal damage. The induced fit of MurK is triggered by binding of MurNAc into the cleft-shaped active site and results in a combined sheered rotation move of the N-terminal domain as a rigid body closing the active site over the phosphate acceptor sugar (Figs. 2*C* and S5). The induced fit of MurK can be visualized by superposing the C-terminal domain of the unliganded MurKapo (“open” conformation) to its corresponding C-terminal domain of MurK/MurNAc (“closed” conformation). The best fit utilizes residue range 106 to 266 and results in a C_α_-RMSD value of 0.67 Å for 152 aligned residues, showing virtually identical backbone conformation of all the aligned residues of that domain (see Fig. 2*C*). The transition from open to closed conformation is based on helix α3, which slides over the C-terminal β-sheet towards the active site, resulting in a rotation by approximately 12° (calculated by DynDom (27), Fig. S5) and a maximum translation of 6.9 Å measured for the C_α_ distance of residue S88 when comparing open and closed states. Beside loops α1β4 and β1β2, of which loop β1β2 pushes towards the sugar-binding site, a C_α_-RMS difference plot of the N-terminal domain from open to closed state would classify the adjustment as a rigid body move as indicated by the total C_α_-RMSD value of 1.1 Å for all residues of the N-terminal domain.

Only minor rearrangements for ATP binding were observed, with an C_α_-RMSD value of 0.78 Å, aligning all residues of the N-terminal domain of MurKapo and MurK in its AMP-PCP–bound state. This is in agreement with the observation that ATP is predominantly held in place by residues of the C-terminal domain. With respect to the dimeric state of the enzyme, the induced fit is also a concentric move of both N-terminal domains towards each other. In the open state, the adjacent protomer does not contribute to the neighboring active site, but the induced fit accompanying the MurNAc uptake enhances the reshaping of the active site by rigidifying the dimeric interface with additional contacts from the N-terminal protomer. This involves contacts made by residues from loop β3α1 and helix α1 (residues 34–39) and residues from loop α1β4 (residues 70,72 und 73) to enlarge the buried surface area of the dimer by almost 500 Å^2^ (Table S2). Similar kinase dynamics on sugar binding are widely reported, for example, the human NagK (22), *Sulfolobus tokodaii* broad-specificity sugar kinase (28), *N-*acetylmannosamine kinases from pathogenic bacteria *Pasteurella multocida* and *Haemophilus influenzae Pm*-NanK, *Hi*-NanK (29), as well as *N-*acetylmannosamine kinases from humans (25). The observation that a strong induced fit takes place only during MurNAc binding is in agreement with the suggested random sequential binding mechanism (21) of sugar kinases. The closed conformation is essential for catalytic turnover, as only in this enzymatic state, the reaction center is shielded and allows for tight interactions with MurNAc.

### The active site and activity of MurK kinase

**Figure 4:**
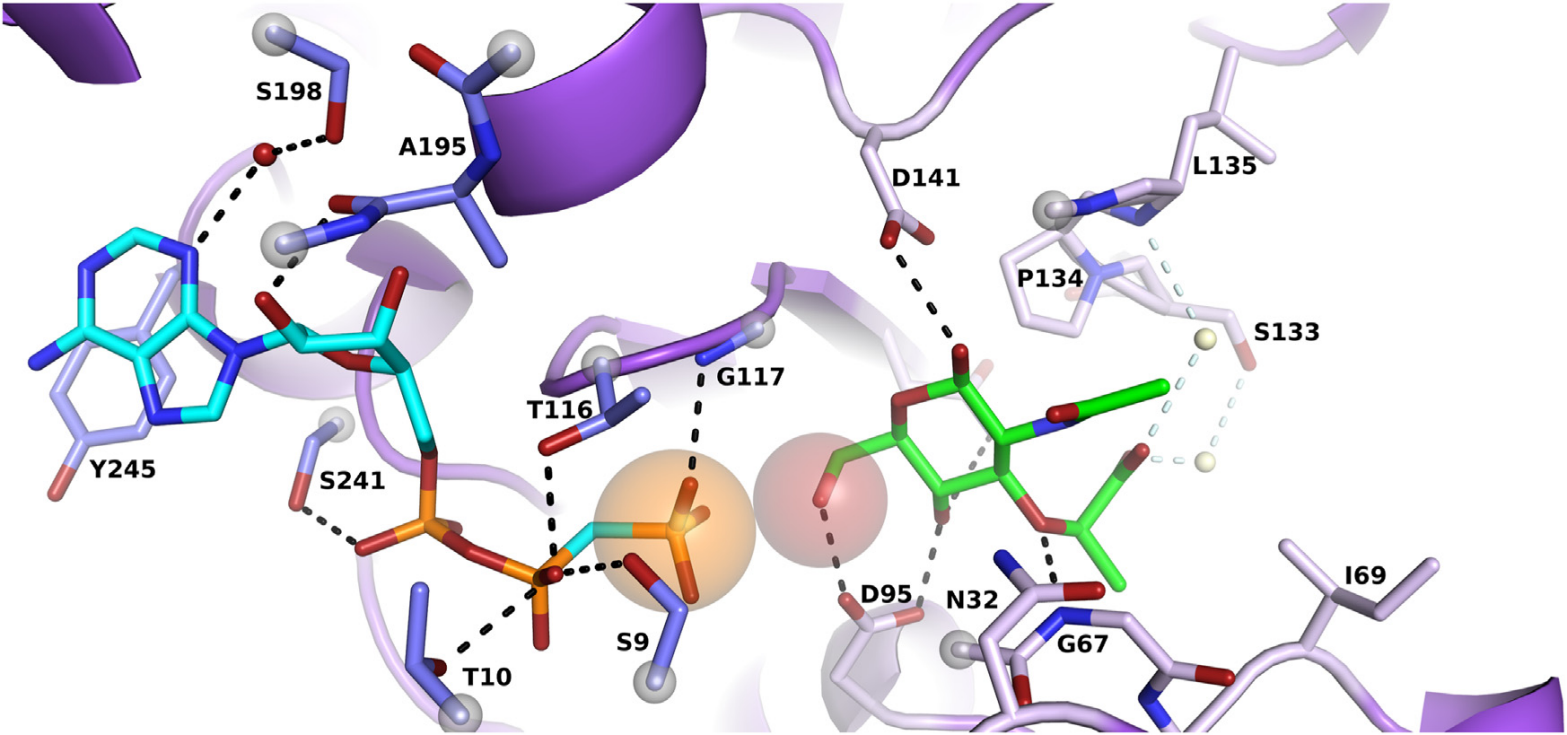
**Structurally informed model of the active site of MurK with both substrates bound.** MurK/AMP-PCP is superimposed on the C-terminal (residues 110–273) of MurK/MurNAc (shown as *cartoon*). Residues involved in binding are shown as *sticks*: *pale purple* for the MurK/MurNAc structure, *blue* for those from the MurK/AMP-PCP structure. *Dashes* represent proposed hydrogen bonds, and *spheres* represent waters (color scheme as in Fig. 3). *Orange* and *red spheres* represent reaction centers for phosphorus and oxygen, respectively. MurK, murein sugar kinase; MurNAc, N-acetylmuramic acid.

The active site is located in a channel between the N- and C-terminal domains of the V-shaped enzyme (Fig. 2*A*). The channel compresses upon MurNAc binding to the enzyme (Fig. 2*B*), likely to enhance the interactions with the sugar molecule. The binding site can be subdivided into two binding regions, the ATP-binding site and the MurNAc-binding site (Figs. 3, *A* and *B* and 4). The ATP-binding site is formed by residues of the loop β6β7 (I113, G115, T116, G117), helix α5 (G145), helices and loops between helices α8 and α11 (A195, G196, S198, P199 and A202, S241, V242, Y244, Y245) on the C-terminal domain. The interactions of the N-terminal domain are limited to residues of the loop β1β2 (G8, S9, T10, and K11), which interact with all phosphoryl groups of ATP (Fig. 3*A*).

The AMP-PCP is clearly bound in both protomers of the ASU of the monoclinic crystals (Fig. S6). Both chains possess an identical conformation (Fig. S7). AMP-PCP is bound at the N-terminal domain *via* a conserved DxGxT motif with interactions of the side chain of S9 upstream of the conserved T10 residue, both forming hydrogen bonds to the β-phosphate group. This common motif is also found in the kinases of *Bacillus subtilis* (30) and *Naegleria fowleri* (31) and, functionally conserved with a serine residue, in human NagK. All other interactions with AMP-PCP are located on the C-terminal domain. T116, also conserved in the homologs, is located on the opposite site of the phosphate entity and forms a hydrogen bond with the β-phosphate of AMP-PCP and the γ-phosphate to the backbone amino group of G117. The adenosine is bound mainly *via* van der Waals interactions contributed by nonpolar residues but forms hydrogen bonds *via* the carbonyl atom of A195 towards the hydroxy group at C2 atom of ribose and a water-mediated hydrogen bond *via* S198 to the adenosine ring system. In addition, the aromatic ring system is aligned coplanar to tightly pack against side chain Y244. Finally, S241, part of a conserved IGSV sequence (residues 238–241), stabilizes the α-phosphate by hydrogen bonding. Magnesium ions have been shown to be important for the function of MurK (11) and other sugar kinases (25, 28). We were not able to unequivocally identify a magnesium ion in any of the MurK structures. Typically, magnesium is found at the terminal phosphate groups to enhance the electrophilic character of the γ-phosphate but also to stabilize the leaving group ADP.

The MurNAc-binding site is located on the opposite site of the ATP molecule and shaped by residues of the loop β1β2 (S9), loop β3α1 (N32, F34), loop β4α2 (G67, I69), and loop β5α3 (D95) on the N-terminal domain. On the C-terminal domain, interactions involve strand β7 (N119), loop α4α5 (D141), and loop β8α4 (S133, P134, L135) (Fig. 3*B*). Structure-based modeling (Fig. 4) suggested that the C6 hydroxy group of MurNAc points towards the terminal phosphate of ATP in the center of the cavity, which is in perfect agreement with the biochemical reaction catalyzed by the enzyme. The anomeric carbon of the glucosamine ring of MurNAc adopts a β-configuration and is held in place by a strictly conserved catalytic residue D95 that establishes hydrogen bonding to the hydroxy groups of C4 and C6. This polarizes the reactive oxygen atom and facilitates the nucleophilic attack on the γ-phosphate group of ATP. In the N-terminal domain, residue S9 and residues of loop β3α1 (N32, F34, F35), of loop β4α2 (A66, G67, C68, I69), and of loop β5α3 and helix α3 (T94, M96) form the MurNAc cavity that is completed by interactions on the C-terminal domain to residues 117 to 119 and the region including residues 133 to 136. These interactions are predominantly van der Waals contacts. Only four residues specifically recognize the functional groups of MurNAc. G67 forms a hydrogen bond to the ether bond of the lactyl ether entity *via* its backbone amide, whereas D95, N119, and D141 utilize their side chains to recognize the hydroxy groups at atom C4 and C6, C4, and C1, respectively (see Fig. 3*B*). The importance of the strictly conserved catalytic D95 is highlighted by its clamp-like interaction with hydroxy groups both at atom C4 and atom C6, a feature that is also reported in other sugar kinases ((22, 32, 33) and PDB ID 8DTC). Both functional groups (*N-*acetyl and lactyl ether) of the MurNAc ring system are not specifically recognized by hydrogen bonding. The *N-*acetyl group dips into a rather hydrophobic cavity that is formed by the backbone of G67 and a set of three phenylalanine side chains (F34, F35, F137). Residue N32, a potential hydrogen bond donor to interact with the carbonyl function of the *N-*acetyl group as reported for the human NagK, is too far away (3.6 Å) to contribute to its recognition but lines the bottom of this cavity. In comparison to NagK, the amide functional group is also not saturated by hydrogen bonding due to amino acid substitution of G145 in NagK with P134 in MurK. The lactyl ether points towards the entrance of the channel. No direct protein interactions are observed to account for the negative charge of the carboxyl group besides shape complementarity of the protein, predominately created by P134 on top of which the lactyl ether sits. When superposing the MurK–AMP-PCP complex to MurK/MurNAc, potential water-mediated interactions of two structurally conserved water molecules with the carboxyl group of MurNAc seem to be likely based on their presence in the high-resolution structure of MurK/AMP-PCP. One of the modeled water molecules bridges the carboxy entity *via* the side chain of S133, whereas the other water molecule would be bonded by an amide backbone interaction to L135.

**Figure 5:**
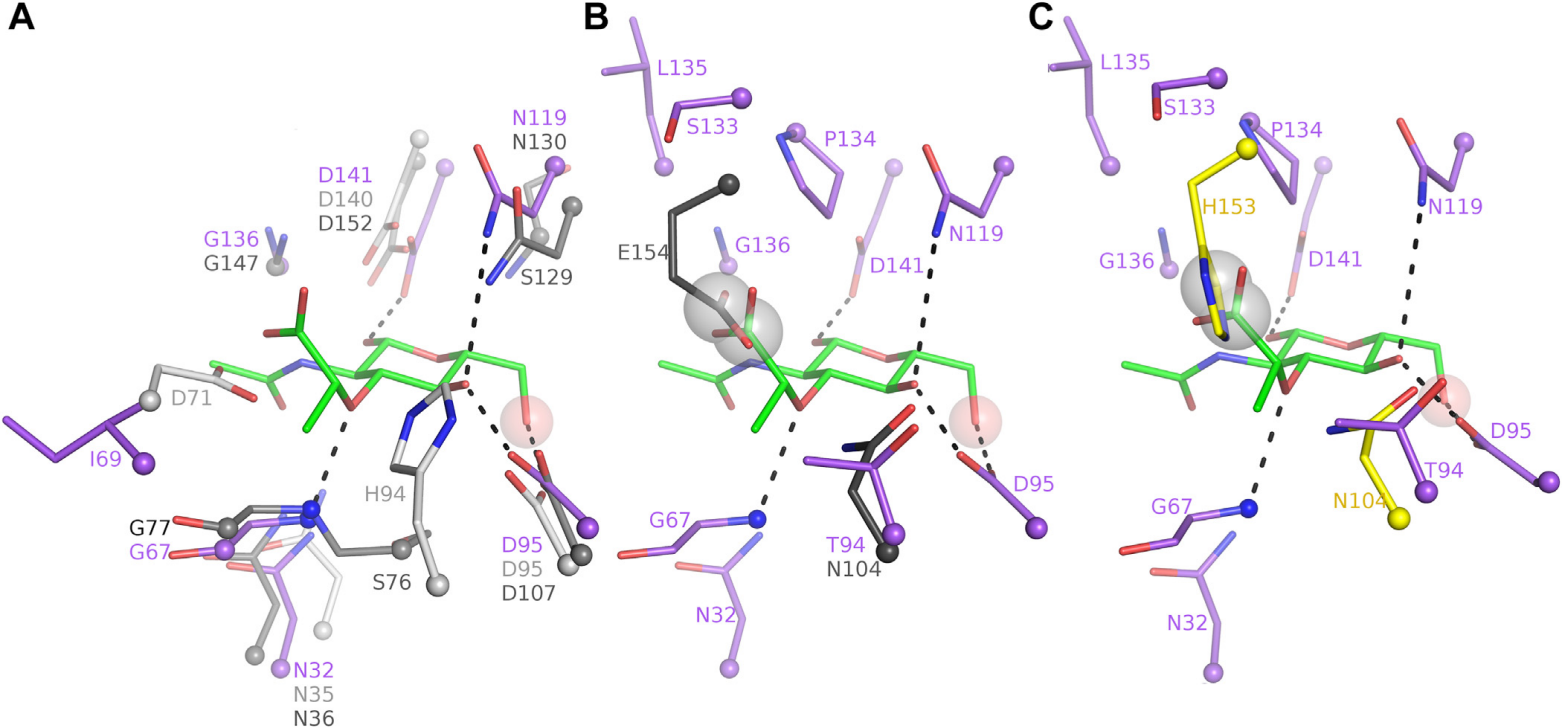
**Sugar-binding residues in the active site of MurK and its structural homologues.***A*, active site residues mediating MurNAc binding in MurK (*purple*), broad-specificity kinase from *Sulfolobus tokodaii* (*white*; PDB ID: 2E2O), and human NagK (*gray*; PDB ID: 2CH6). Structures are superimposed on the sugar (*green*). *B*, active site residues mediating MurNAc binding or MurNAc specificity in MurK (*purple*) and those causing clashes in *Streptomyces griseus* glucokinase (*black*, PDB ID: 3VGL). *C*, active site residues mediating MurNAc binding or MurNAc specificity in MurK (*purple*) and those causing clashes in *Haemophilus influenzae* mannosamine kinase (*yellow*, PDB ID: 6JDB). Structures in all panels were superimposed only on the sugar molecules present in the active sites to demonstrate the similarities and differences in sugar binding. MurK, murein sugar kinase; MurNAc, N-acetylmuramic acid; NagK, N-acetylglucosamine kinase.

We compared the binding mode of available hexokinases and ROK enzyme structures by superposing the conserved catalytic aspartate residues from different proteins (Fig. 5). These included the human NagK (22), the hexokinase from *S. tokodaii* (28), human hexokinase I (32), the inorganic polyphosphate/ATP-glucomannokinase from *Arthrobacter* sp. *strain KM* (33), the fructokinase from *B. subtilis* (30), the ROK glucokinase from *Streptomyces griseus* (34), glucokinase from *N. fowleri* (31), the glucokinase from *Acanthamoeba castellanii* (PDB ID: 8DTC), the *N-*acetylmannosamine kinase from *H. influenzae* (29), and the glucokinase from *Trypanosoma cruzi* in complex with a GlcNAc-linked inhibitor (PDB ID: 7S2P). It is noteworthy that all glucose molecules or glucose derivatives adopt a virtually identical chair conformation with β-configuration at the anomeric C-atom when bound to their corresponding enzymes, with the exception of GlcNAc binding to NagK (22), which adopts an α-configuration.

When comparing MurNAc kinases to glucose or GlcNAc kinases, analysis indicates the reason for MurNAc specificity to be related to two positions (Fig. 5). Most obviously, residues S133, P134, and L135 in MurK allow for MurNAc binding by providing sufficient space for the lactyl ether substituent. Except for the human NagK (22) and the hexokinase from *S. tokodaii* (28), the corresponding position in the homolog enzymes listed above carries a bulky residue that is either charged (mostly a glutamate residue) or polar (H153, (29)) that would sterically clash with lactyl ether entity, abolishing the binding of MurNAc (see Fig. 5). A second position crucial for MurNAc recognition arises from residue T94. Here, the homolog sugar kinases carry a polar side chain (asparagine, except for hexokinase from *S. tokodaii*, where a histidine residue is found; Fig. 5) that is involved in binding the hydroxy group at C4 of the sugar molecule. This interaction is not observed for MurK and would result in steric stress towards the lactyl ether substituent, abolishing MurNAc binding. In hexokinase from *S. tokodaii*, the sterical clash to the lactyl ether moiety is moreover reinforced by substitution of I69D which would additionally interfere with MurNAc binding *via* charge repulsion.

The reaction pathway can be inferred from superposing both substrate structures, MurK/MurNAc and MurK/AMP-PCP (see Fig. 4). The terminal phosphate group of ATP is in close proximity to the hydroxy group of MurNAc, with a distance of 3.4 Å to the phosphorus atom that is attacked during the reaction. The clamp-like binding by D95 polarizes the hydroxyl group to facilitate a nucleophilic engagement of the oxygen atom at C6 of MurNAc. Such a polarization would facilitate a nucleophilic attack on the terminal phosphate group of ATP. During catalysis, D95 probably acts as a catalytic base to abstract the proton of the nascently generated MurNAc-6P. The acid-base catalysis of this type of sugar phosphorylation has been previously confirmed by site-directed mutagenesis, for example, human kinase I almost completely loses catalytic activity (99% activity loss) when the catalytic aspartate residue is replaced by an alanine or the human mannosamine kinase loses catalytic activity on mutating the aspartate to alanine or asparagine (25, 35, 36).

**Figure 6:**
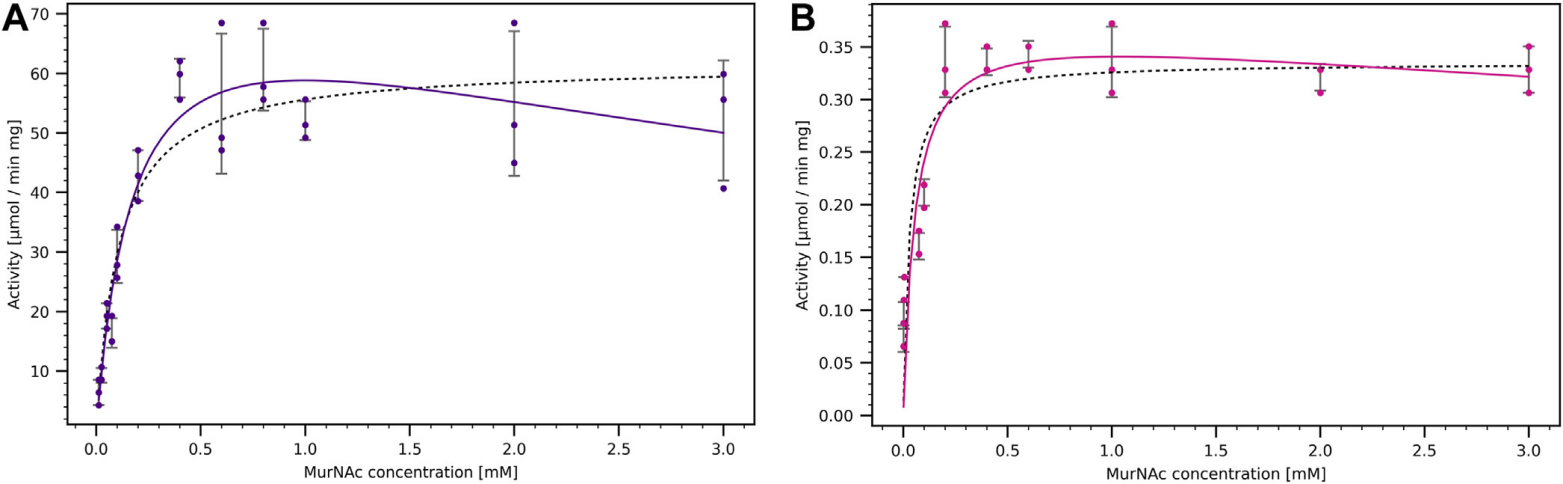
**Kinetics ofMurKand K1058for thephosphorylation of MurNAc**. The activity depending on MurNAc concentration for MurK (*A*) and K1058 (*B*) is shown. The *black dashed line* is fitted to the Michaelis–Menten model, and the *purple* (MurK) or *magenta* (K1058) line is fitted to the substrate-inhibited model. All measurements were performed in triplicate, and each point represents the fastest reaction rate observed in the UV spectra. Where only two data points are visible per concentration, the values of two points were overlapping. MurK, murein sugar kinase; MurNAc, N-acetylmuramic acid.

Based on the structural data, we reinvestigated the enzymatic activities that were reported for MurK acting as MurNAc kinase with a K_M_ value of 113 μM and a catalytic turnover of k_cat_/K_M_ of 69,910 M^−1^s^−1^ with little acceptance of the enzyme for GlcNAc (11). Our activity measurements are in agreement with previous measurements (Table 2) and show an affinity towards MurNAc with similar apparent K_M_ values (110 ± 20 μM for strict Michaelis–Menten kinetics and 180 ± 40 μM taking substrate inhibition into account). The substrate-inhibition model fit the data better (Fig. 6), a result that was also reported in the studies by Hottman *et al.* (11). The structural basis for substrate inhibition remains to be determined.

**Table 2:** Apparent kinetic parameters for MurK and K1058 with MurNAc as a substrate

Protein	K_M_ [μM]	v_max_ [μmol min^−1^ mg^−1^]	k_cat_ [s^−1^]	K_i_ [mM]	k_cat_/K_M_ [M^−1^ s^−1^]
MurK	110 ± 20	62 ± 2.7	32 ± 1.4	-	300,000 ± 56,000
MurK∗	180 ± 40	80 ± 8.0	42 ± 4.2	5.6 ± 2.3	230,000 ± 56,000
K1058	28 ± 10	0.34 ± 0.02	0.18 ± 0.01	-	6400 ± 2900
K1058∗	55 ± 22	0.38 ± 0.04	0.20 ± 0.02	20 ± 23	3600 ± 1500

Both the Michaelis–Menten and substrate-inhibited models (marked with ∗) were fitted into the experimental data.

### Crystal structure and activity of MurK paralog K1058

Based on genomic sequence analysis, a second putative MurNAc kinase, termed K1058, was identified in *T. forsythia*, sharing a sequence similarity of 44% to MurK. In an attempt to establish the structural differences between the two MurKs, the K1058 ortholog was cloned and the protein heterologously expressed and purified in a similar procedure as described for MurK. We determined the structure of K1058 to establish the structural differences (Figs. 3, *C* and *D* and S8). We were able to obtain structures of the apo enzyme (K1058apo) and K1058 in complex with MurNAc (K1058/MurNAc) (Fig. S2). A superposition showed a close relationship between K1058 and MurK, with an observed C_α_-RMSD value of 2.1 Å over the total protein length. An in-depth active site comparison of both MurNAc-liganded structures showed a very similar binding of MurNAc as in MurK, with the sugar molecule slightly tilted around the bond between O1 and C5 (Fig. 3, *C* and *D*). When comparing all residues of the sugar-binding site, differences can be found in two locations. Firstly, residue I69 of MurK is replaced by a less bulky and more polar threonine (T69) in K1058. Secondly, in the region involved in lactyl ether recognition, residues S133 and P134 are substituted by an arginine and an alanine, respectively. All other residues in the MurNAc-binding site are identical and occupy conformations very close to MurK (Fig. 3*D*). We speculate that the substitutions should not severely structurally influence the affinity of MurNAc binding to K1058. The residues I69 and P134 in MurK give an excellent shape complementarity, which corresponds to residues T69 and R132 in K1058, respectively. Hence, structural data would not suggest a decrease in MurNAc affinity. Moreover, R132 in K1058 seems to be an optimal candidate to bind MurNAc *via* its carboxy group. Although we do not observe such an interaction, probably because the side chain conformation would have to be in an “bent back” setup, a partial charge compensation is possible, which might compensate for the loss in affinity by the loss of water-mediated hydrogen bonding to S133 in MurK. We confirmed our observations by activity measurements with K1058 (Table 2). Indeed, the affinity for K1058 is higher than MurK, with an apparent K_M_ value of 28 ± 10 μM, but the catalytic turnover is severely reduced and suggests that MurNAc might not be the natural substrate of K1058. To exclude enzyme aggregation as the reason for this observation, we performed CD spectroscopy and analytical SEC experiments (Fig. S9) on K1058. These revealed folded protein and did not show significant aggregation even on incubation of the enzyme for 4 days at 4 °C and after freezing and thawing. A reason for this unexpected observation is still to be determined, but we speculate that the subtle changes in MurNAc binding are mainly caused by the exchanges at the positions S133 and P134 in MurK to R132 and A133 in K1058. The backbone geometry is rigidified by P134, resulting in a tighter packing of this region towards MurNAc and therefore favors MurNAc binding to MurK in comparison to K1058. In summary, the enzymatic assays showed that K1058 has low efficiency for MurNAc compared to MurK, although its affinity is higher. One possible explanation for the unusual kinetics of K1058 would be that the enzyme needs to be (allosterically) activated for full efficiency. Currently, we would assume that the natural substrate of K1058 is not MurNAc, rather it may act as an inhibitor. A speculative explanation for the observation of high affinity but low turnover of MurNAc could be a controlling mechanism to downregulate K1058 processing of the natural substrate in the presence of MurNAc.

## Discussion

Structural analyses of MurK revealed that the enzyme belongs to the group of ribonuclease H-like kinases, with a fold that is very similar to that of other sugar kinases, particularly those classified as ROK kinases (despite MurK not belonging to that family based on sequence). Ribonuclease H-like kinases show a remarkable similarity across domains of life, with the top matches for structural similarity being an uncharacterized bacterial protein (PDB IDs: 1ZXO) and a putative NagK (PDB code: 1ZBS), followed by a broad-specificity glucokinase from the hyperthermophilic archeon *S. tokodaii* (28) and the human NagK in complex with GlcNAc (22). The broad structural conservation of sugar phosphorylation is unsurprising, given its role in basic metabolic functions such as glycolysis. Our work presents the first structure of a MurNAc kinase, which broadens the general understanding of specificity for sugars within the kinase family. The MurK structure enabled us to identify residue patterns in the protein that are crucial to accommodate MurNAc. Based on these patterns, we are now able to determine whether a hexokinase of interest is capable of binding MurNAc or MurNAc derivatives. Although we cannot identify MurNAc binding from sequence analysis, MurNAc binding can be inferred from the vastly increasing amounts of structural data based on *in silico* folding approaches by AlphaFold2 (37). Our analysis of currently available experimental data of sugar kinases in complex with their substrates suggests that the majority of deposited hexokinases are not able to use MurNAc as a substrate due to steric clashes with bulky polar or bulky charged residues at positions corresponding to region 133 to 136 or at positions corresponding to T94 of MurK. On the other hand, the structure-based MurNAc recognition pattern enables us to hypothesize that both proteins from structural genomic consortium projects (1ZXO and 1ZBS) are kinases at least able to phosphorylate MurNAc, if MurNAc is not their natural substrate. Another example concurring with the MurNAc specificity regions is NagK. Comparing the structural data of NagK with MurK indicates no conflict for MurNAc binding and would suggest that NagK is able to accommodate MurNAc as a potential phosphate acceptor, although NagK initially was characterized as a GlcNAc kinase. Indeed, recent studies by Stafford *et al.* (16) identified NagK as being essential for the immunostimulatory activity of muramyl dipeptides by their phosphorylation at C6 of the MurNAc entity. The reaction is comparable to MurK and in excellent agreement with the specificity-controlling positions obtained from structural data of MurK. This work therefore broadens our view of how hexokinases structurally achieve their specificities for their conjugate substrates.

The catalytic turnover of MurK was previously investigated *via* TLC assays (11). The experiment showed that the kinase is capable of phosphorylating both MurNAc and GlcNAc in an extended assay with an incubation time of 16 h, and MurK showed a clear preference for MurNAc. When comparing K1058 and MurK with GlcNAc kinases, for example, human NagK (22), a structural reason for GlcNAc specificity cannot be concluded from the available data on both enzymes. Noteworthy, the only obvious difference in binding the *N-*acetyl entity of GlcNAc seems to be related to a tryptophan residue (W38 in human NagK), which is functionally replaced by a phenylalanine residue in both MurK and K1058. The preference for MurNAc over GlcNAc therefore seems to be the binding of the lactyl ether moiety.

The data presented here enable the structure-based development of MurNAc analogs as specific inhibitors of MurNAc kinases. These could be helpful to differentiate their cellular functions and to evaluate their potential as drug targets in organisms relying on such enzymes, particularly in combination with other metabolic targets. A similar approach using GlcN-derivatized inhibitors such as benzoyl glucosamine or hydroxyphenyloxopropyl glucosamine aims to block the *Leishmania* glucose kinases from *Leishmania braziliensis* for the treatment of leishmaniasis (38) or, for example, carboxybenzyl glucosamine to specifically inhibit the glucokinase and hexokinase from *T. cruzi*, a pathogen causing Chagas disease (39). The approach is promising, resulting in growth inhibition IC_50_ values in the low micromolar range (39).

In conclusion, we determined the first structure of a MurNAc kinase, MurK, from *T. forsythia*, a pathogen of the red complex associated with the late stages of periodontitis. We showed by kinetic analysis that both MurK and K1058 are capable of phosphorylating MurNAc, although with different enzymatic affinities and catalytic turnover rates. The natural substrate of K1058 remains to be determined. A structure comparison with other glucokinase and hexokinases from different sources showed relaxed steric restraints at the lactyl ether entity, which serves as a basis for the specificity of MurNAc preference. Our findings broaden our understanding of how kinases evolved substrate specificity and might lead to a structure-guided development of new MurNAc analogs to specifically block MurNAc-dependent kinases of pathogenic bacteria for antibiotic treatment.

## Experimental procedures

### Protein expression and purification

The cloning of MurK has been described previously (11), and the K1058 construct was generated according to the same protocol. Briefly, an 855-bp DNA fragment containing the *T. forsythia* K1058 gene (*Tanf_RS08300)* was amplified by PCR from the bacterial genome using the forward primer 5′GCGCCATGGCGAAATTAATAGCAGAAAGCGGATCAACG3′ and reverse primer 5′GCGCTCGAGGCTTTCCGGATCATCCGGAGGGAAGGC3′. The PCR product and target vector pET28a (Novagen) were digested with NcoI and XhoI (New England Biolabs) restriction enzymes and ligated into pET28a with T4 DNA ligase (Thermo Fisher Scientific). To obtain large amounts of high purity proteins for crystallization trials, MurK and K1058 were expressed in *E. coli* BL21(DE3) cells. First, chemically competent cells were transformed by heat shock with plasmid DNA (approx. 100 ng) coding for the desired protein genes. Next, an overnight culture of LB medium (120 ml) containing 50 μg/ml kanamycin (all chemicals Sigma-Aldrich, unless otherwise stated) was inoculated with either a freshly transformed cell suspension or with cells from a glycerol stock. The overnight culture was then used for large scale protein production in LB-Kanamycin medium (2 L or 4 L). Here, cells were grown at 37 °C to an A_600_ of 1.1, then expression was induced by addition of IPTG (final concentration of 0.5 mM), while the temperature was reduced to 18 °C. The cells were harvested by centrifugation after 20 h of incubation and either stored at −80 °C or directly used for purification of the target protein. All purification steps were conducted at 4 °C. The cells were resuspended in immobilized metal ion affinity chromatography (IMAC) wash buffer (25 mM Tris–HCl, 500 mM NaCl, 25 mM imidazole, 10% (v/v) glycerol, pH 8.0). Cell lysis was performed by sonication. The lysate was clarified by centrifugation at 34,500*g* for 45 min, and the resulting supernatant was loaded onto a His-trap column (5 ml; GE Healthcare) pre-equilibrated with IMAC wash buffer. The column was then installed onto an ÄKTA prime system and washed with IMAC wash buffer, and IMAC wash buffer was supplemented with 5% and 10% of the elution buffer (as IMAC wash buffer, with 500 mM imidazole) until UV-monitoring showed that all impurities and unspecific binders were removed. The target proteins were eluted with 80% (v/v) elution buffer and fractionated based on inspection of UV absorbance at 280 nm. The resulting fractions were analyzed by SDS-PAGE and pooled by purity. The solution was concentrated (molecular weight cut-off of 10 kDa) by centrifugation (Amicon, Merck). Typically, 4 to 6 ml of the target protein solution was obtained at concentration between 2 to 6 mg/ml. The protein was loaded onto an SD200 16/60 SEC column equilibrated with SEC buffer MurK (200 mM NaCl, 50 mM MES, pH 6) or SEC buffer K1058 (750 mM NaCl, 25 mM Tris-HCl, pH 8.5) and eluted as 2 ml fractions. These were analyzed by SDS-PAGE, and pure protein fractions were pooled and concentrated. Typical yields were 12 mg for MurK and 5 to 10 mg for K1058 per 1 L culture. Analytical SEC was performed on an SD200 Increase 3.2/300 column pre-equilibrated with the appropriate SEC buffer by loading 50 μl of the sample and eluting it with the same buffer.

### Crystallization experiments

Initial screens were performed in a 96-well plate format as sitting drop experiments at two different temperatures (4 °C and 20 °C) using either a Tecan Freedom Evo 150 (Tecan) or as Gryphon pipetting robot (Art Robins Instruments). The protein solution was mixed in a 1:1 ratio with the corresponding reservoir solution and placed over the reservoir. Initial crystallization hits from commercial screens (Molecular Dimensions; Hampton Research) were optimized for crystal quality. For K1058, the best crystals were obtained in a solution of ammonium sulfate (0.02–0.08 M; Roth), MES monohydrate (0.1 M, pH 6.3–6.9), and PEG 5000 (16%–21% (w/v)), whereas MurK crystallized in bis-tris propane/citric acid mixture (0.1 M, pH 4.5), ammonium sulfate (0.3 M), and PEG3350 (16% (w/v)). Crystals grew within a few days to weeks in both cases.

As our attempts of soaking the apo crystals were unsuccessful due to crystal packing issues, we performed cocrystallization experiments using nonhydrolyzable substrate analogs AMP-PNP (adenylyl-imidodiphosphate; Roche) or AMP-PCP and MurNAc or GlcNAc and preincubated for 30 min with the kinases to obtain substrate-bound kinase crystals. The substrate solutions were dissolved in the appropriate SEC buffer at concentrations of 100 mM (sugar substrates) or 20 mM (phosphate analogs). MurNAc, which would precipitate in the MurK SEC buffer due to the pH, was dissolved in 1 M Hepes, pH 7.5, instead. The substrate solutions were added in a 1:10 ratio to the purified kinases, resulting in final concentrations of 2 to 10 mM for the crystallization trials. We obtained MurK–AMP-PCP complex crystals in 0.1 bis-tris propane/citric acid mixture at pH 5.5, 0.12 to 0.14 M ammonium sulfate, and 14 to 18% w/v PEG3350 within weeks, while the K1058–MurNAc complex could be crystallized after 6 months in 1.5 M ammonium sulfate (Roth), 0.1 M Tris pH 8.5, 12% (v/v) glycerol, and the MurK–MurNAc complex after 9 months in 15% PEG 10000, 100 mM trisodium citrate pH 5.5, 2% v/v 1,4-dioxane. MurK/MurNAc crystals resulted from cocrystallization and grew within 6 months to maximum dimensions of 60 × 25 × 5 μm^3^ in an apparent space group I4_1_22 as a result of almost perfect merohedral twinning.

### X-ray diffraction experiments and structure solution

The crystals were cryoprotected by harvesting into a fresh solution of the crystallization condition with the addition of a cryoprotectant solution (20%–30% (v/v) glycerol, except for K1058/MurNAc, where 15% (v/v) glycerol was used based on (40)), mounted, and flash-frozen in liquid nitrogen for data collection at the synchrotron. Data was collected at beamline I03 at Diamond Light Source (Oxford) or at beamline X06 Da at the Swiss Light Source . Data reduction was performed using the XDS/XSCALE package (41). The phase problem for the first structure (MurKapo) was solved by molecular replacement using PDB entry with accession code 1ZBS after adjustments to the sequence using CHAINSAW (42) and to the structure coordinates using PYMOL (43). The MurK/AMP-PCP and K1058 structures were solved using MurKapo structure for molecular replacement, while for K1058/MurNAc, K1058apo was the model and K1058/MurNAc was used for MurK/MurNAc (see below). Initial models were refined using a simulated annealing approach as implemented in PHENIX (44) to reduce model-induced bias. The models were then completed using several cycles of real space model corrections in COOT (45), followed by reciprocal space refinement as implemented in REFMAC5 (46). Solvent molecules were placed in several cycles by a combination of the COOT:Find_waters algorithm followed by refinement with REFMAC5. Unequivocal positive density was observed in a simulated annealing (F_o_-F_c_)-difference omit map for the three ligand-bound structures before the appropriate ligands were placed into it.

Due to merohedral twinning, the MurK/MurNAc structure was the most challenging case. The crystal structure of MurK/MurNAc was solved in space group I4 using K1058/MurNAc as a template with the sugar removed to avoid introducing bias and the C- and N-terminal domains placed individually in the crystal lattice. Further refinement involved simulated annealing with PHENIX.refine using the model target geometry of MurKapo to restrain the refinement. The simulated annealing omit map density unequivocally showed the presence of MurNAc in four out of six protomers in a virtually identical position as observed for K1058/MurNAc. The density of the remaining two protomers is generally of lower quality probably due to crystal disorder caused by twinning. The final steps of refinement for K1058, MurK, and MurK/AMP-PCP included Translation-Libration-Screw-rotation parameterization as analyzed by TLSMD (47). During the final steps of refinement, substrate molecules were placed based on a simulated annealing (F_o_-F_c_)-difference omit map and subsequently refined with REFMAC5.

All models were validated with tools implemented in COOT and the PDB validation server (48). Figures were generated with PYMOL, and coordinates and structure factor amplitudes were deposited in the PDB under ID codes 8QQW, 8QQX, 8OW9, 8QQK, 8OW7 for MurK, MurK in complex with AMP-PCP, MurK in complex with MurNAc, K1058, and K1058 in complex with MurNAc, respectively.

### CD spectroscopy

To verify the folding of K1058, we used CD spectroscopy on a Jasco J-720 spectropolarimeter. We investigated freshly thawed K1058 enzyme that was used for enzymatic assays. To determine the folding stability, we incubated the sample for 4 days at 4 °C and repeated the measurement. The measurements were performed at a protein concentration of 0.3 mg/ml, in a glass cuvette with a light pathway of 1 mm. CD spectra were recorded by ten repeated measurements in a wavelength range of 195 nm to 250 nm. These measurements were averaged and subtracted from the buffer measurements. The resulting CD spectra were analyzed by BETSEL (49).

### Enzymatic activity assays

The apparent kinetic parameters of MurK and K1058 for the phosphorylation of MurNAc with ATP were determined by a coupled enzyme assay (20). In this assay, the formation of ADP was directly, stoichiometrically coupled to NADH oxidation by pyruvate kinase and lactate dehydrogenase. In a 400 μl QS cuvette with a 10 mm light path, 50 mM Hepes (pH 7.5, Roth), 10 mM MgCl_2_ (Merck), 5 mM ATP, 1 mM phosphoenolpyruvate, 0.2 mM NADH, 100 U/ml of pyruvate kinase, and 70 U/ml of lactate dehydrogenase (from rabbit muscle, 0.1 U of pyruvate kinase/μl and 0.07 U of lactate dehydrogenase/μl) were incubated with MurNAc at final concentrations ranging from 0.0125 or 0.025 to 3 mM for MurNAc. For every individual measurement, we used freshly thawed enzyme (MurK or K1058) from the same batch of protein preparation to maintain reproducibility. The hydrolysis of ATP was initiated by the addition of 178 ng of MurK and 17.6 μg K1058. The final volume of the reaction was 400 μl: 320 μl master mix, 40 μl protein (8.9 mg/ml diluted to 1:2000 with 1 M Hepes pH 7, 178 ng per reaction), and 40 μl MurNAc at 10 times the final concentration diluted in 50 mM Hepes pH 7. The reactions were then incubated at 20 °C for 45 min or until no change in absorbance was observed. The change of NADH absorbance was monitored at 340 nm in a spectrophotometer (WTW Photometer PhotoLab 7600 UV-Vis, Xylem Analytics). Apparent kinetic parameters were evaluated using matplotlib as implemented in python by fitting the experimental data to the Michaelis–Menten equation and Michaelis–Menten kinetics including substrate inhibition. We used a molar extinction coefficient of NADH at 340 nm of 6.22 × 10^3^ M^−1^ cm^−1^ for calculation of the V_max_ and k_cat_ values.

## Data availability

The coordinates and structure factors for the X-ray structures have been deposited in the Protein Data Bank under accession codes 8QQW, 8QQX, 8OW9, 8QQK, 8OW7 for MurK, MurK in complex with AMP-PCP, MurK in complex with MurNAc, K1058, and K1058 in complex with MurNAc, respectively.

## Supporting information

This article contains supporting information (26, 50, 51).

## Conflicts of interest

The authors declare that they no conflicts of interest with the contents of this article.
