# Supplementary material for: N-acetylmuramic acid recognition by MurK kinase from the MurNAc auxotrophic oral pathogen Tannerella forsythia
Source: J Biol Chem. 2023 Jul 20;299(9):105076. doi: 10.1016/j.jbc.2023.105076 (PMC10465942; doi:10.1016/j.jbc.2023.105076)
Supplement: Supporting Figures S1–S9 and Tables S1–S5 [file mmc1.docx]

Supporting information

*N-*acetylmuramic acid recognition by MurK kinase from the MurNAc auxotrophic oral pathogen *Tannerella forsythia*

**Authors:**

Aleksandra Cecylia Stasiak^1^, Karolin Gogler^1^, Marina Borisova^2^, Phillipp Fink^1^, Christoph Mayer^2^, Thilo Stehle^1^, and Georg Zocher^1*^

^1^ Interfaculty Institute of Biochemistry, University of Tuebingen, D-72076 Tuebingen, Germany

^2^ Interfaculty Institute of Microbiology and Infection Medicine, Organismic Interactions / Glycobiology, University of Tuebingen, D-72076 Tuebingen, Germany

* Corresponding author: Dr. Georg Zocher (Interfaculty Institute of Biochemistry, University of Tuebingen, 72076 Tuebingen, Germany, e-mail: [georg.zocher@uni-tuebingen.de](mailto:georg.zocher@uni-tuebingen.de), phone: +49 7071 2974762)

Running title: Structural analysis of MurNAc kinases

Keywords: ATPase, bacterial metabolism, cell wall, cell wall recycling, MurNAc kinase, periodontal disease, protein structure


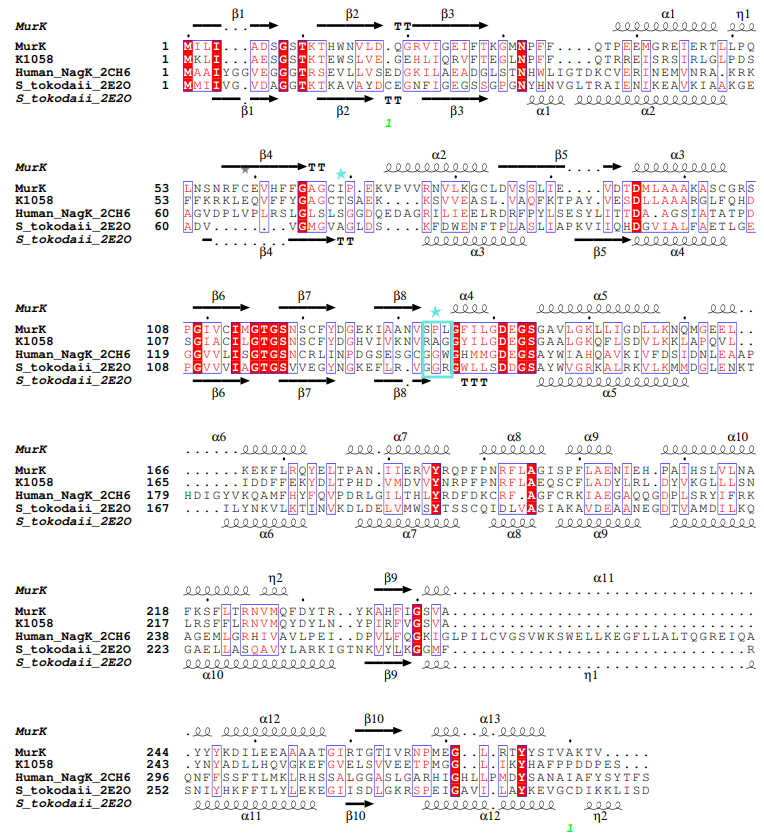
**Supplementary Figure S1. Sequence alignment of MurK and K1058 with two closest structural homologues with known functions**. The human N-acetylglucosamine kinase NagK (PDB ID: 2CH6) and the archaeal S. tokodaii broad-specificity sugar kinase (PDB ID: 2E2O). Cyan border and star indicate residues important for MurNAc recognition. Alignment generated with MUSCLE (50), figure with ESPRIPT (51).


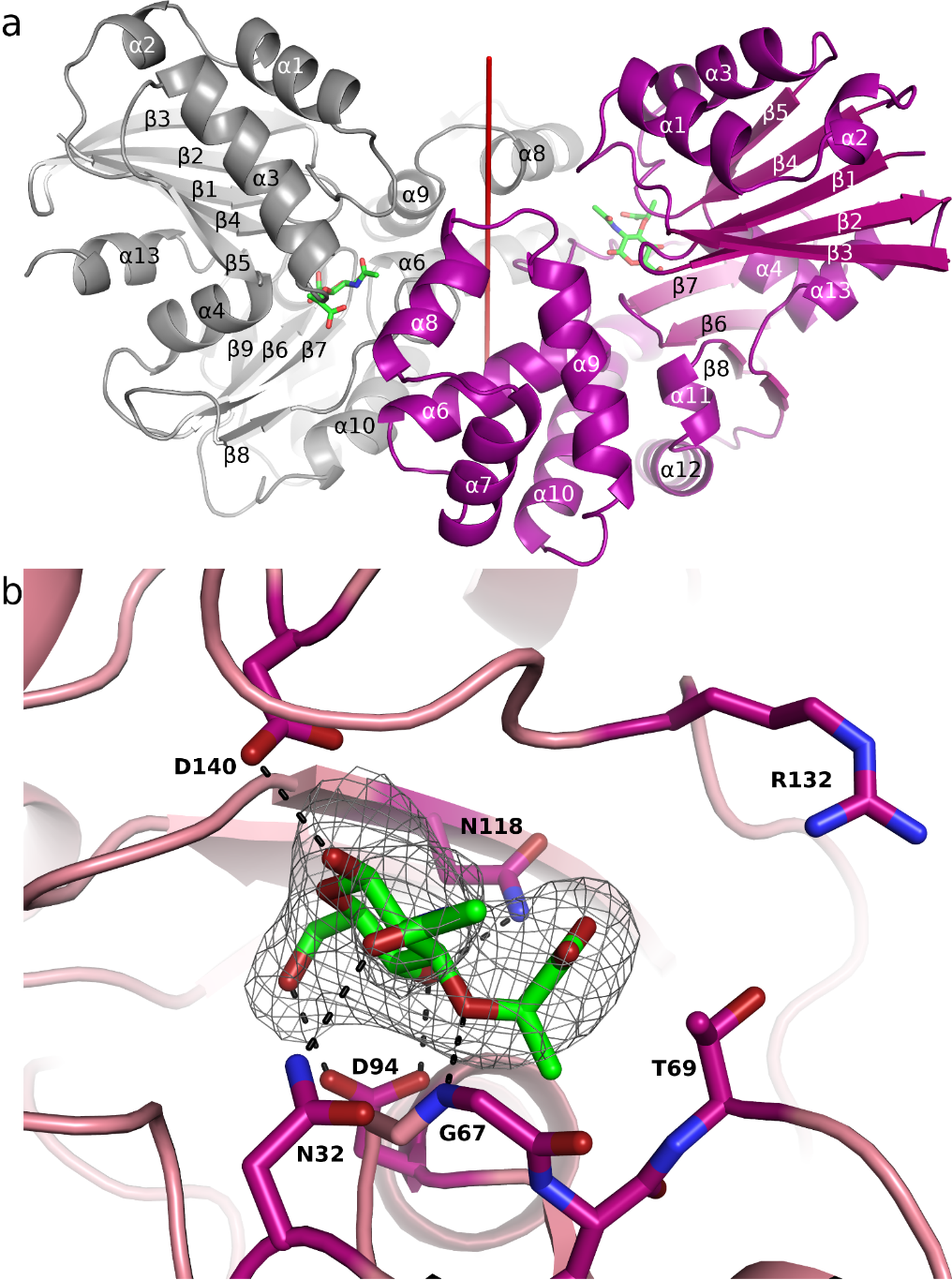


**Supplementary Figure S2. The structure of K1058**. Cartoon representation of the biological K1058 dimer (one chain in magenta, one in grey) in complex with the MurNAc substrate (green). The red line shows the two-fold symmetry axis.


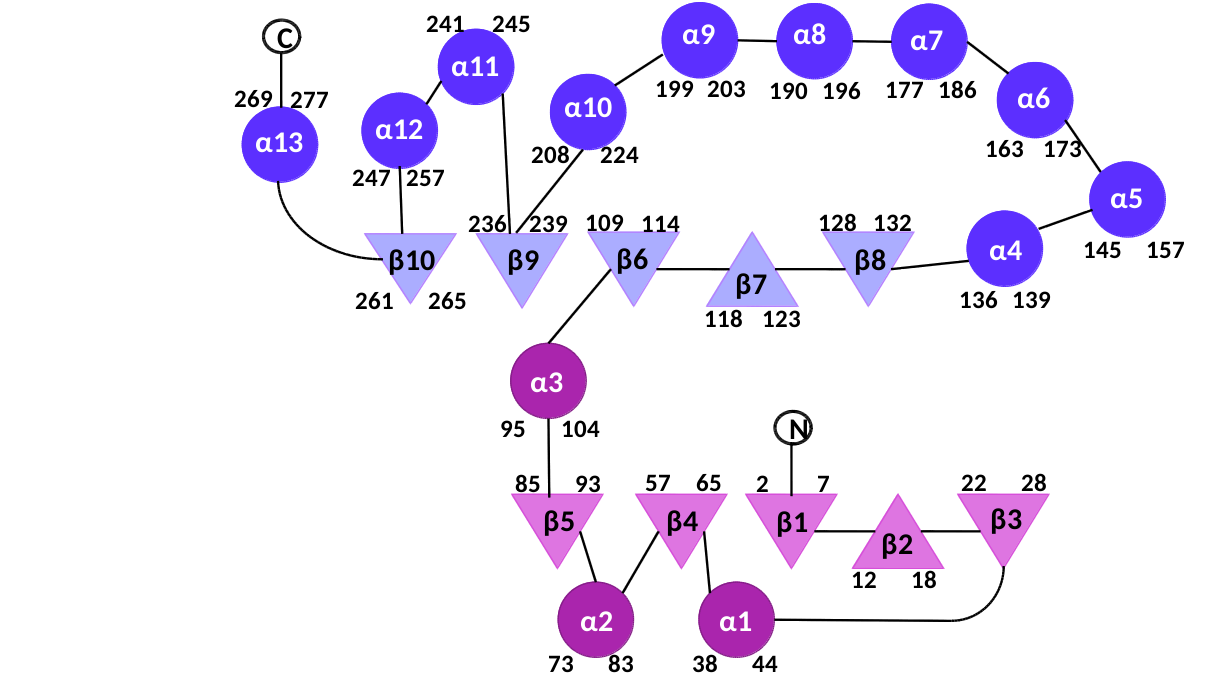


**Supplementary Figure S3. Topology plot of MurK**. N-terminal domain residues in magenta, C-terminal domain residues in violet.


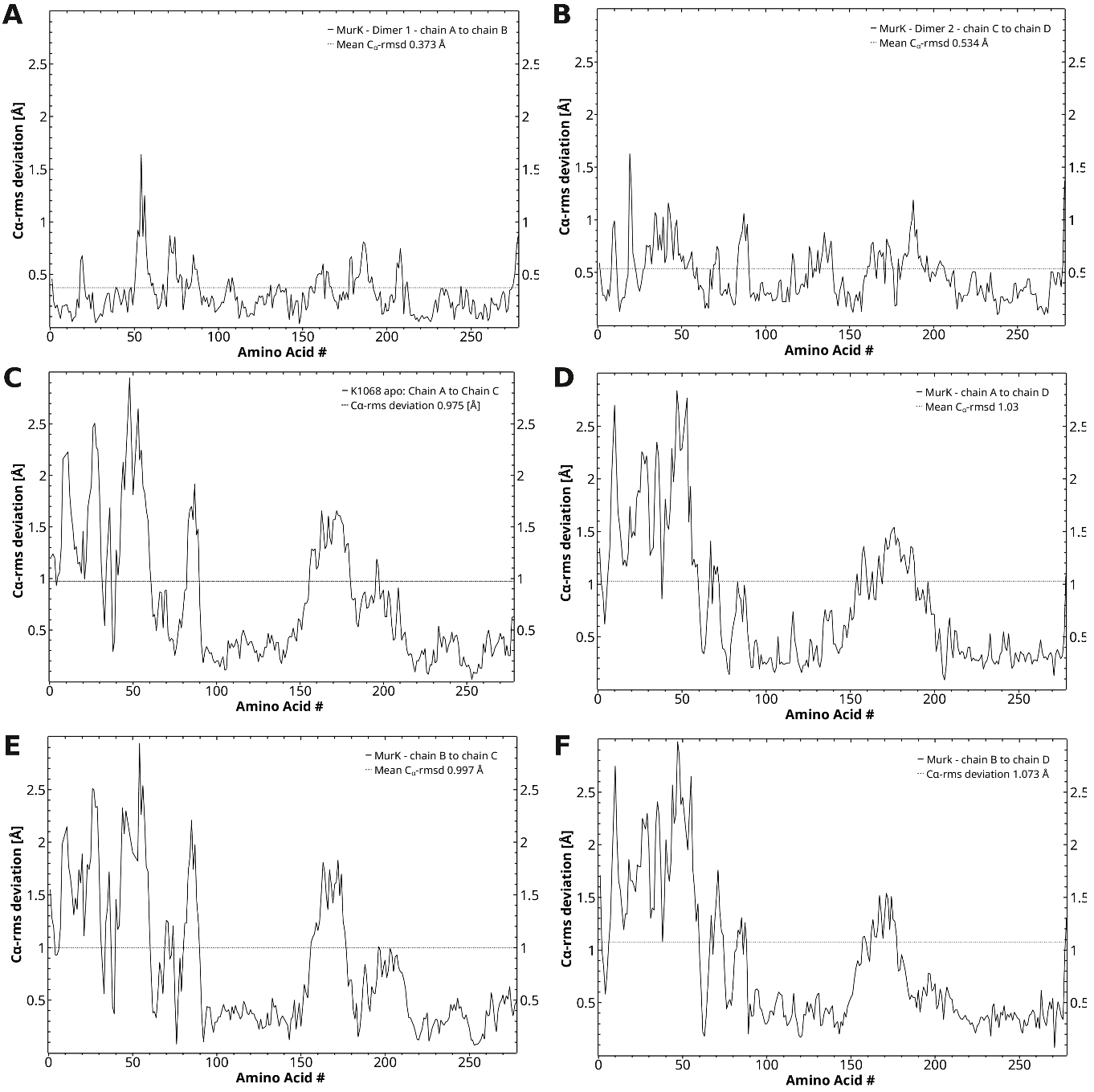


**Supplementary Figure S4. Structural differences of the individual chains in the crystal structure of MurK**. The triclinic crystal contains four protomers in the unit cell. Each chain was superposed on all others (A to F) and its corresponding amino acid C_α_-RMSD was plotted. Both protomers in a biological dimer (chains A, B and chains C, D) possess a virtually identical conformation, as indicated by a very low mean C_α_-RMSD. Comparing the chains of a dimer to the adjacent dimer showed a different conformation by increased C_α_-RMSD values, especially in the flexible N-terminal domain and residues 160 to 180 of the C-terminal domain.


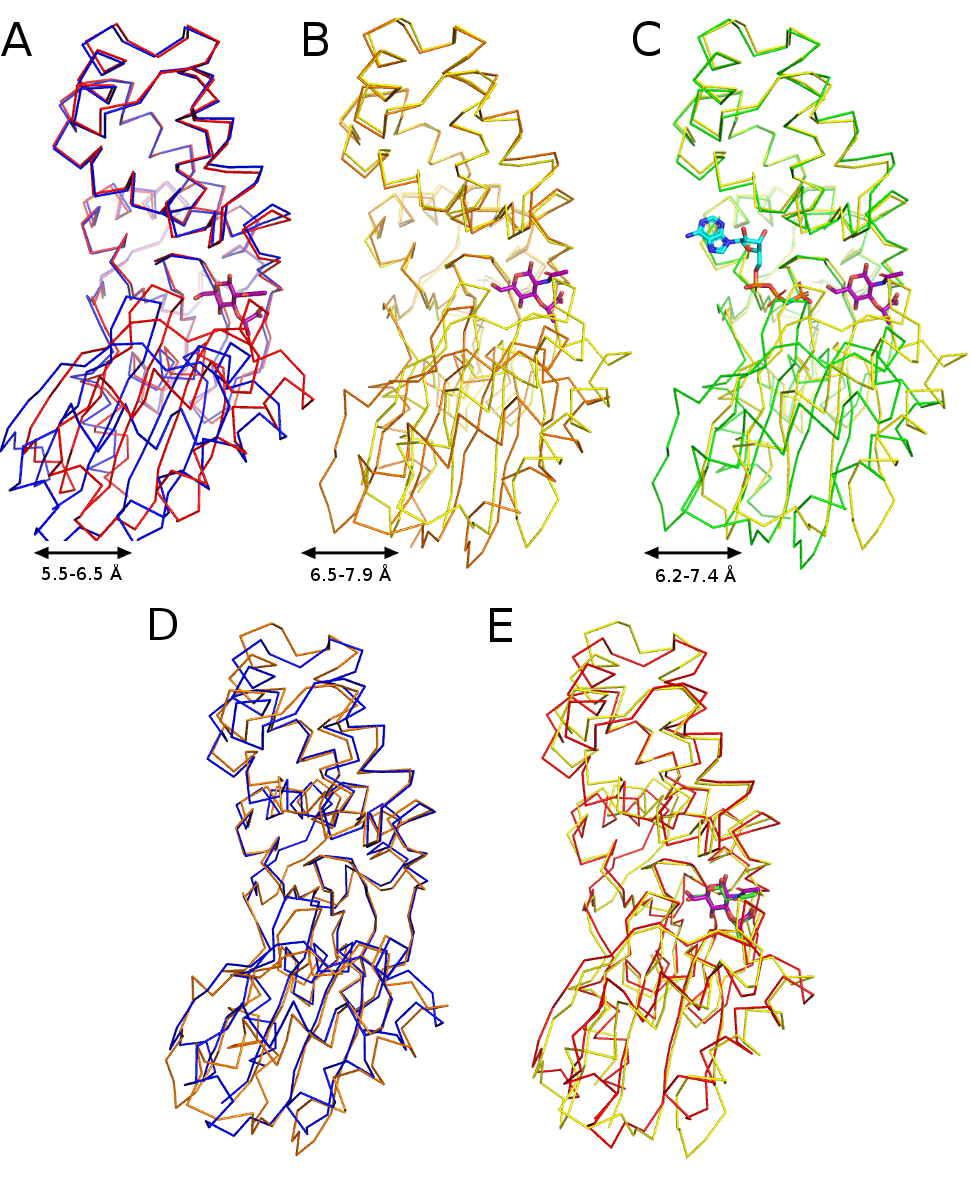


**Supplementary Figure S5. N-terminal domain movement on MurNAc binding in MurK and K1058**. (A) K1058 apo (blue) and K1058/MurNAc (red), superimposed to residues 91-272. (B) MurK apo (orange) and MurK/MurNAc (yellow), superimposed to residues 91-273. (C) MurK/AMP-PCP (green) and MurK/MurNAc (yellow), superimposed to residues 91-273. (D) MurK apostructure (orange) and K1058 apostructure (blue), superimposed on the C-terminal domains. (E) MurK/MurNAc (yellow) and K1058/MurNAc (red), superimposed on C-terminal domains.


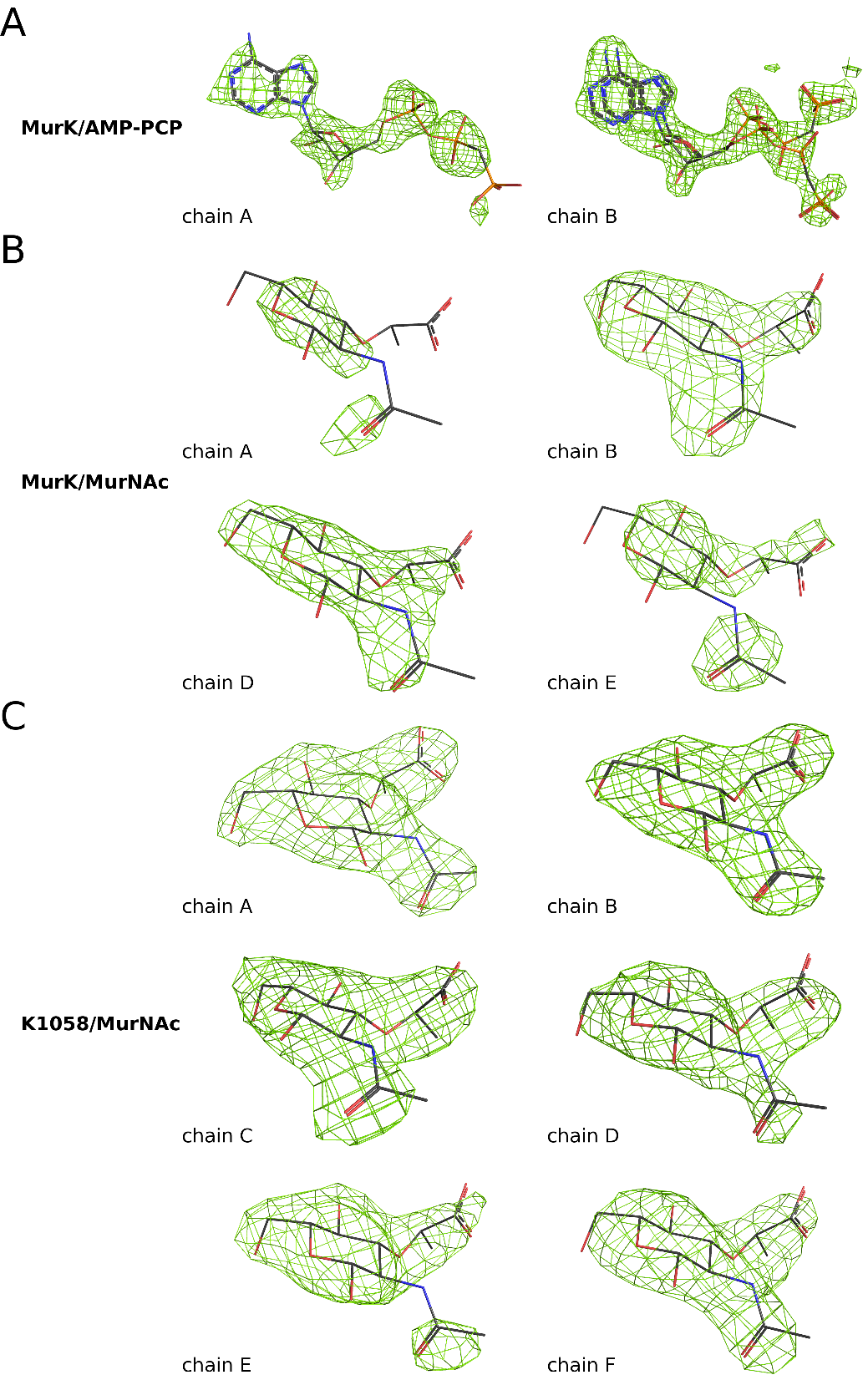


Supplementary Figure S6. **Substrate densities in the complex structures of MurK and K1058**. Unbiased simulated annealing difference electron density omit maps (green) at a contour level of 2.5σ or 3.0σ for AMP-PCP **(A)** or MurNAc in MurK/MurNAc and K1058/MurNAc **(B and C)**, respectively. Except for MurK/MurNAc, the electron density unequivocally shows the presence of the corresponding ligand in all chains of the asymmetric units. For the twinned crystal MurK/MurNAc the electron density is weak for two chains in general (chain C and F) and does not allow placing MurNAc in these chains. The density is acceptable for chain A and E, where MurNAc was placed. In chains B and D, the electron density unequivocally shows the presence of the substrate MurNAc and its conformation in the active site which is similar as observed for K1058/MurNAc.


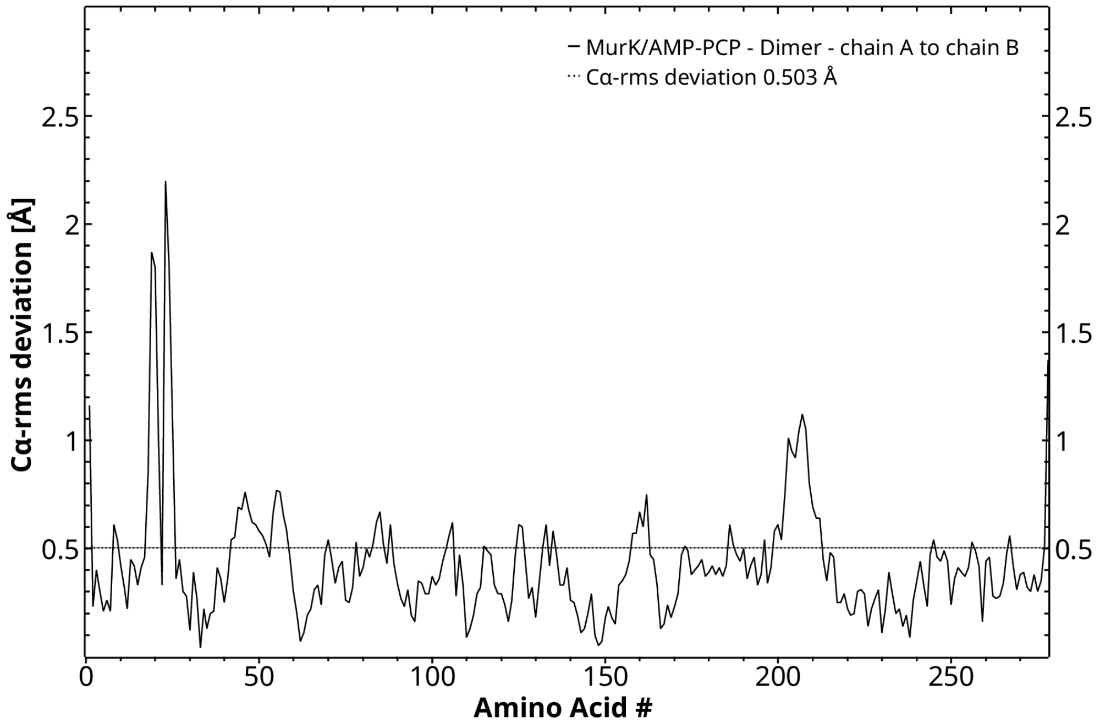


Supplementary Figure S7. **Structural differences of the individual chains in the crystal of MurK/AMPPCP**. The monoclinic crystal contains a biological dimer in the asymmetric unit. Chain A was superposed to chain B and the corresponding C_α_-RMSD was plotted. Both protomers possess a virtually identical conformation with a mean C_α_-RMSD of 0.5 Å.

*
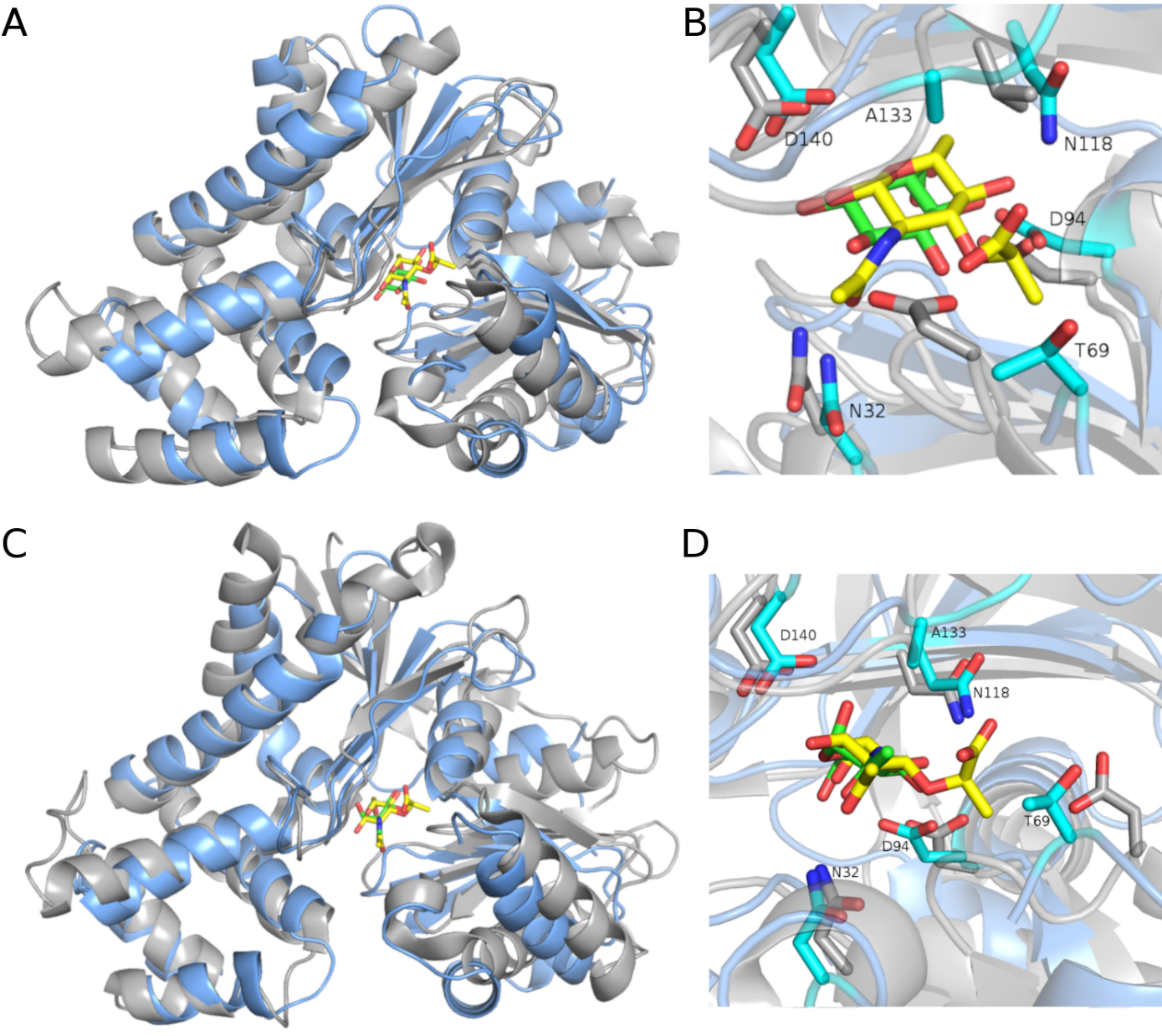
*

**Supplementary Figure S8.** **Structural comparison of K1058**. (A) cartoon representation of K1058 (blue) superimposed with the broad-specificity glucokinase from *S. tokoidaii* (2E2O; grey) on the C-terminal. (B) Close-up of the active site from (A), with active site residues of K1058 labelled. (C) cartoon representation of K1058 (blue) superimposed with the human NagK kinase (2CH6; grey). (D) Close-up of the active site from (C), with active site residues of K1058 labelled.


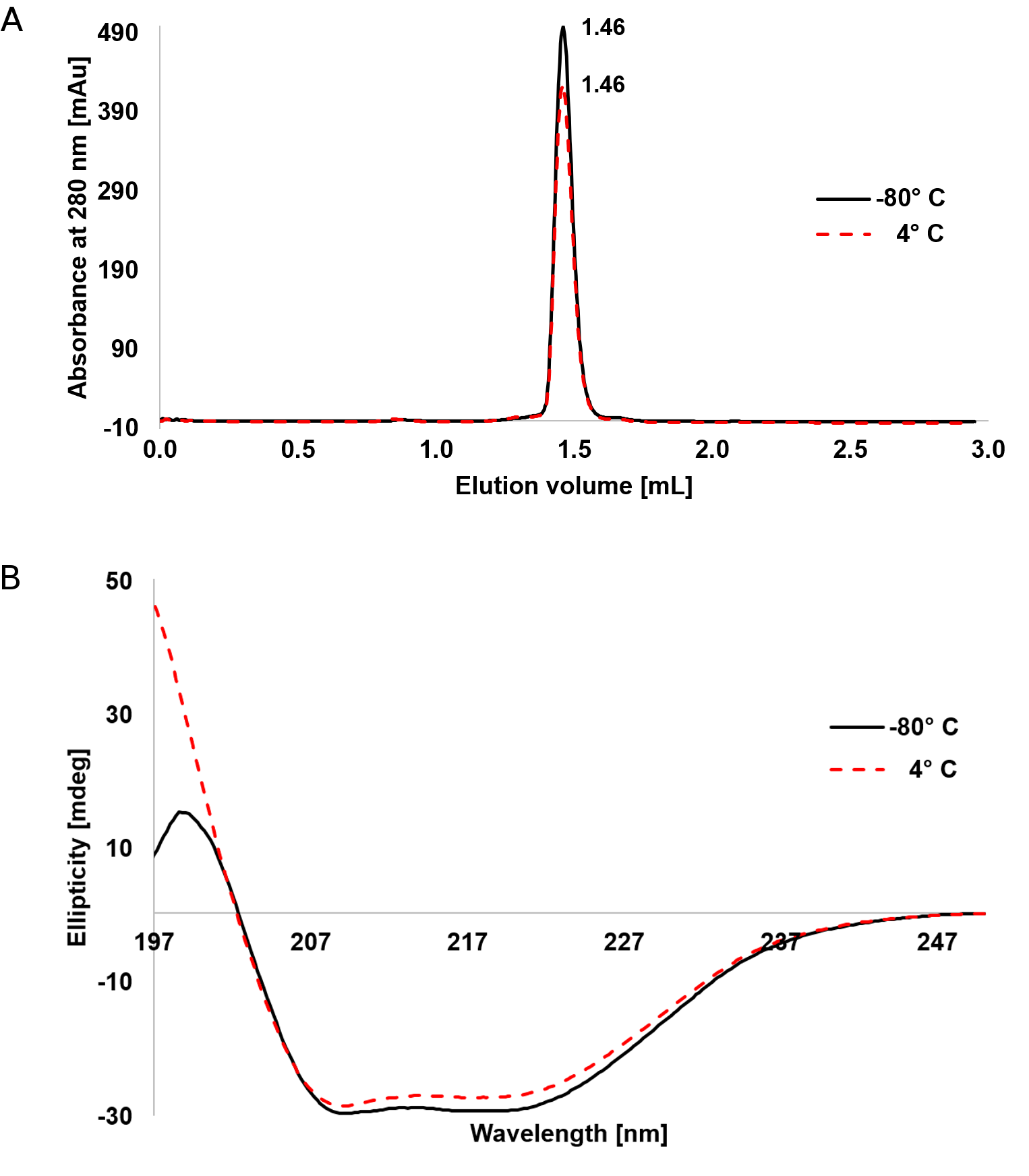


**Supplementary Figure S9. Stability experiments and CD spectroscopy of K1058**. (A) Kinase stability was investigated by analytical SEC using a SD200 increase 3.2/300 column. Frozen-thawed enzyme is compared to a sample that was stored at 4°C for four days. Although the intensities decreased within 4 days by 16%, no evident formation of aggregates could be observed. The elution volume of 1.46 mL corresponds to an apparent molecular mass of 60 kDa. (B) Both protein samples were investigated by CD spectroscopy to verify their folding. Although no severe stability issues were observed all enzymatic assay data were obtained from freshly thawed enzyme. Analysis of the CD spectra of both samples revealed an α-helical content of 36.7% and an antiparallel β-strand content of 18.7% for the freshly thawed K1058. After 4 days at 4 °C only subtle changes were detected yielding to an α-helical content of 35.6% and an antiparallel β-strand content of 12.9%.

**Supplementary Table S1**. Percentage sequence similarity between MurK and K058 and their closest characterised structural homologues in Supplementary Figure 1.

| Protein | MurK | K1058 | Human_NagK_2CH6 | S_tokodaii_2E2O |
| --- | --- | --- | --- | --- |
| MurK | 100.00 | 44.24 | 17.50 | 22.22 |
| K1058 | 44.24 | 100.00 | 16.73 | 17.82 |
| Human_NagK_2CH6 | 17.50 | 16.73 | 100.00 | 23.81 |
| S_tokodaii_2E2O | 22.22 | 17.82 | 23.81 | 100.00 |

**Supplementary Table S2**. List of residues involved in the formation of the dimeric interface of MurK identified by PISA. The solvent accessible area (SAA), the buried surface area (BSA) and the fraction of the total surface area of the individual amino acid (AA) are given. Only residues with a percentage buried surface area over 80% are listed.

| Structure | Chain:Residue | SAA [Å^2^] | BSA [Å^2^] | Fraction of BSA per AA |
| --- | --- | --- | --- | --- |
| MurK APO | A:ARG 21 | 138 | 121 | 88% |
| MurK APO | A:PHE 27 | 110 | 104 | 95% |
| MurK APO | A:THR 48 | 47 | 40 | 84% |
| MurK APO | A:GLN 172 | 75 | 63 | 85% |
| MurK APO | A:TYR 173 | 59 | 57 | 97% |
| MurK APO | A:PHE 193 | 24 | 19 | 82% |
| MurK APO | A:ILE 197 | 8 | 8 | 100% |
| MurK APO | B:ARG 21 | 135 | 112 | 83% |
| MurK APO | B:PHE 27 | 110 | 102 | 93% |
| MurK APO | B:THR 48 | 34 | 29 | 86% |
| MurK APO | B:TYR 173 | 53 | 52 | 99% |
| MurK APO | B:ILE 197 | 8 | 8 | 100% |
| MurK APO | B:SER 277 | 38 | 30 | 81% |

**Supplementary Table S3**. List of residues involved in the formation of the dimeric interface of MurK/AMPPCP identified by PISA. The SAA, the BSA and the fraction of the total surface area of the individual amino acid (AA) are given. Only residues with a percentage buried surface area over 80% are listed.

| Structure | Chain:Residue | SAA [Å^2^] | BSA [Å^2^] | Fraction of BSA per AA |
| --- | --- | --- | --- | --- |
| MurK AMPPCP | A:LEU 135 | 111 | 97 | 88% |
| MurK AMPPCP | A:ILE 138 | 124 | 114 | 91% |
| MurK AMPPCP | A:LEU 139 | 133 | 123 | 92% |
| MurK AMPPCP | A:ILE 153 | 14 | 13 | 96% |
| MurK AMPPCP | A:GLY 154 | 34 | 32 | 96% |
| MurK AMPPCP | A:LYS 158 | 155 | 153 | 99% |
| MurK AMPPCP | A:VAL 184 | 27 | 25 | 91% |
| MurK AMPPCP | A:LEU 194 | 3 | 3 | 100% |
| MurK AMPPCP | B:LEU 135 | 112 | 96 | 86% |
| MurK AMPPCP | B:ILE 138 | 126 | 115 | 91% |
| MurK AMPPCP | B:LEU 139 | 130 | 119 | 91% |
| MurK AMPPCP | B:ILE 153 | 14 | 13 | 95% |
| MurK AMPPCP | B:GLY 154 | 36 | 34 | 96% |
| MurK AMPPCP | B:LYS 158 | 155 | 154 | 99% |
| MurK AMPPCP | B:ILE 180 | 2 | 2 | 93% |
| MurK AMPPCP | B:VAL 184 | 27 | 25 | 93% |
| MurK AMPPCP | B:LEU 194 | 3 | 3 | 100% |

**Supplementary Table S4**. List of residues involved in the formation of the dimeric interface of MurK/MurNAc identified by PISA. The SAA, the BSA and the fraction of the total surface area of the individual amino acid (AA) are given. Only residues with a percentage buried surface area over 80% are listed.

| Structure | Chain:Residue | SAA [Å^2^] | BSA [Å^2^] | Fraction of BSA per AA |
| --- | --- | --- | --- | --- |
| MurK MurNAc | A:PRO 33 | 14 | 14 | 100% |
| MurK MurNAc | A:PHE 34 | 109 | 95 | 86% |
| MurK MurNAc | A:LEU 135 | 113 | 98 | 87% |
| MurK MurNAc | A:ILE 138 | 130 | 114 | 88% |
| MurK MurNAc | A:LEU 139 | 131 | 117 | 90% |
| MurK MurNAc | A:ILE 153 | 14 | 14 | 95% |
| MurK MurNAc | A:GLY 154 | 32 | 31 | 96% |
| MurK MurNAc | A:LYS 158 | 142 | 142 | 100% |
| MurK MurNAc | A:ILE 180 | 7 | 7 | 98% |
| MurK MurNAc | A:VAL 184 | 30 | 26 | 85% |
| MurK MurNAc | A:LEU 194 | 7 | 7 | 100% |
| MurK MurNAc | A:ASN 225 | 4 | 4 | 100% |
| MurK MurNAc | B:PRO 33 | 20 | 18 | 91% |
| MurK MurNAc | B:PHE 34 | 107 | 87 | 82% |
| MurK MurNAc | B:LEU 135 | 115 | 104 | 91% |
| MurK MurNAc | B:ILE 138 | 133 | 122 | 92% |
| MurK MurNAc | B:LEU 139 | 127 | 111 | 87% |
| MurK MurNAc | B:ILE 153 | 14 | 14 | 94% |
| MurK MurNAc | B:GLY 154 | 32 | 31 | 97% |
| MurK MurNAc | B:LYS 158 | 147 | 147 | 100% |
| MurK MurNAc | B:ILE 180 | 10 | 9 | 90% |
| MurK MurNAc | B:VAL 184 | 33 | 30 | 90% |
| MurK MurNAc | B:TYR 185 | 171 | 138 | 81% |
| MurK MurNAc | B:LEU 194 | 6 | 6 | 100% |
| MurK MurNAc | B:ASN 225 | 6 | 6 | 100% |

**Supplementary Table S5**. Highest-scoring DALI (26) matches for MurK and K1058 apo structures, sorted by Z-score.

| Name | Organism | PDB Code | Z-score | RMSD [Å] | Similarity to |
| --- | --- | --- | --- | --- | --- |
| Conserved hypothetical protein Q8A1P1 | *Bacteroides thetaiotaomicron* | 1zxo | 40.5 | 1.1 | MurK |
| Putative N-acetylglucosamine kinase (PG1100) | *Porphyromonas gingivalis* | 1zbs | 40.3 | 1.1 | MurK |
| N-acetylglucosamine kinase (NAGK) | Human | 2ch5 | 23 | 2.9 | MurK |
| Broad-specificity hexokinase | *Sulfolobus tokodaii* | 2e2o | 23 | 3 | MurK |
| Putative N-acetylglucosamine kinase | *Chromobacterium violaceum* | 1zc6 | 22.8 | 3.1 | MurK |
| Cell shape-determining protein MREB | *Spiroplasma eriocheiris* | 7e1g | 17.9 | 2.7 | MurK |
| N-acetylmannosamine kinase | *Haemophilus influenzae* | 6jdb | 17 | 3.5 | MurK |
| Glucokinase | *Streptomyces griseus* | 3vgl | 16.6 | 3.2 | MurK |
| α-skeletal actin | Rabbit | 2yjf | 15.6 | 3.3 | MurK |
| Conserved hypothetical protein Q8A1P1 | *Bacteroides thetaiotaomicron* | 1zxo | 39.1 | 1.2 | K1058 |
| Putative N-acetylglucosamine kinase (PG1100 | *Porphyromonas gingivalis* | 1zbs | 38.1 | 1.5 | K1058 |
| Broad-specificity hexokinase | *Sulfolobus tokodaii* | 2e2o | 22.9 | 2.9 | K1058 |
| Putative N-acetylglucosamine kinase | *Chromobacterium violaceum* | 1zc6 | 22.9 | 2.8 | K1058 |
| N-acetylglucosamine kinase (NAGK) | Human | 2ch5 | 22.4 | 3 | K1058 |
| N-acetylmannosamine kinase | *Haemophilus influenzae* | 6jda | 17.1 | 2.9 | K1058 |
| Cell shape-determining protein MREB | *Spiroplasma eriocheiris* | 7e1g | 17.1 | 2.7 | K1058 |
| Glucokinase | *Streptomyces griseus* | 3vgl | 16.8 | 3.5 | K1058 |
